# Transcription Factor *Mavib-1* Negatively Regulates Conidiation by Affecting Utilization of Carbon and Nitrogen Source in *Metarhizium acridum*

**DOI:** 10.3390/jof8060594

**Published:** 2022-06-01

**Authors:** Xueling Su, Hong Liu, Yuxian Xia, Yueqing Cao

**Affiliations:** 1 School of Life Sciences, Chongqing University, Chongqing 401331, China; xuelingsu@haut.edu.cn (X.S.); 202126021033@cqu.edu.cn (H.L.); 2 Chongqing Engineering Research Center for Fungal Insecticides, Chongqing 401331, China; 3 Key Laboratory of Gene Function and Regulation Technologies under Chongqing Municipal Education Commission, Chongqing 401331, China

**Keywords:** entomopathogenic fungi, transcription factor *Mavib-1*, conidiation, carbon and nitrogen utilization

## Abstract

Conidium is the main infection unit and reproductive unit of pathogenic fungi. Exploring the mechanism of conidiation and its regulation contributes to understanding the pathogenicity of pathogenic fungi. *Vib-1*, a transcription factor, was reported to participate in the conidiation process. However, the regulation mechanism of *Vib-1* in conidiation is still unclear. In this study, we analyzed the function of *Vib-1* and its regulation mechanism in conidiation through knocking out and overexpression of *Vib-1* in entomopathogenic fungus *Metarhizium acridum*. Results showed that the colonial growth of *Mavib-1* disruption mutant (Δ*Mavib-1*) was significantly decreased, and conidiation was earlier compared to wild type (WT), while overexpression of *Mavib-1* led to a delayed conidiation especially when carbon or nitrogen sources were insufficient. Overexpression of *Mavib-1* resulted in a conidiation pattern shift from microcycle conidiation to normal conidiation on nutrient-limited medium. These results indicated that Mavib-1 acted as a positive regulator in vegetative growth and a negative regulator in conidiation by affecting utilization of carbon and nitrogen sources in *M. acridum*. Transcription profile analysis demonstrated that many genes related to carbon and nitrogen source metabolisms were differentially expressed in Δ*Mavib-1* and OE strains compared to WT. Moreover, *Mavib-1* affects the conidial germination, tolerance to UV-B and heat stresses, cell wall integrity, conidial surface morphology and conidial hydrophobicity in *M. acridum*. These findings unravel the regulatory mechanism of *Mavib-1* in fungal growth and conidiation, and enrich the knowledge to conidiation pattern shift of filamentous fungi.

## 1. Introduction

The insect pathogenic fungi *Metarhizium* spp. and *Beauveria* spp. have been widely used all over the world and play an important role in the control of agricultural pests [1,2,3,4,5,6,7]. Representative species, such as *Metarhizium anisopliae, Metarhizium acridum* and *Beauveria bassiana*, have been developed as environmentally friendly biopesticides [8,9]. Entomopathogenic fungi mainly infect host insects through conidia, which are produced under complex regulation. Clarification of the regulation mechanisms of conidiation is also helpful for understanding their invasion and growth inside host insects.

Most fungi have two types of conidiation: normal conidiation (NC) and microcycle conidiation (MC) [10]. Normally, conidiophores arise from the vegetative mycelia and produce large amounts of conidia [11,12], which is the most common conidiation type for filamentous fungi [13]. The fungi can bypass the mycelia period in MC and develop secondary conidia from germ tubes or directly from conidial cells [1,4,14]. MC is a special survival mechanism of fungi under adverse conditions, such as high temperature [10,11,15], extreme pH [16], high salt content [17], and nutritional deficiencies [4,18,19,20]. Among them, nutritional deficiency is the important factor that affects the fungal growth and development.

A previous investigation showed that *M. acridum* produces conidia by NC on nutrient-rich medium 1/4 SDAY (Sabouraud dextrose medium with yeast extract) and MC on the nutrient-deficient medium SYA (sucrose yeast extract agar) [4]. However, adding a single carbon source (sucrose) or nitrogen source (sodium nitrate) in SYA medium can make the *M. acridum* switch to the NC type, indicating that nutrient element plays important roles in the conidiation pattern shift in *M. acridum* [20]. The underlying regulation mechanisms of changes from conidiation to vegetative growth when nutrients are changed are still largely unknown. A *Vib-1* gene (vegetative incompatibility blocked) was highly upregulated when sucrose was added to SYA medium, indicating a possible role of *Vib-1* in conidiation and conidiation pattern shift by affecting the nutrient utilization in *M. acrdium*.

Vib-1 belongs to the Ndt80/PhoG DNA binding protein family and contains a conserved DNA-binding domain NDT80_PhoG. In *Neurospora crassa*, *Vib-1* participates in the regulation of heterokaryotic incompatibility and conidiation [21], and also regulates the expression of cell wall-degrading enzymes (PCWDEs) [22]. Vib-1 acts as a main regulator of the response to carbon and nitrogen starvation. Loss of *Vib-1* leads to the imbalance of Carbon Catabolite Repression (CCR), which inhibits glucose signaling and CCR under carbon-limited conditions in *N. crassa* [23]. In *Aspergillus nidulans*, *Vib-1* is required for production of extracellular proteases in response to carbon and nitrogen starvation [23,24]. However, the roles of *Vib-1* in entomopathogenic fungi and its regulation mechanism in conidiation and fungal growth are not clear.

On SYA medium, the change of nutrients can directly lead to a switch between vegetative growth and conidiation, that is, switching between MC and NC. Therefore, in this study, we used SYA medium to analyze the effect of *Mavib-1* on fungal development under different nutritional conditions in *M. acridum* through gene disruption and overexpression techniques. Exploring the function of transcription factor *Mavib-1* and its mechanism of conidiation pattern shift in *M. acridum* will help to further understand the molecular mechanism of nutrient-regulated fungal conidiation mode shift.

## 2. Materials and Methods

### 2.1. Fungal Strains and Growth Conditions

*M. acridum* CQMa102 was used as a wild-type strain (WT) and stored in the China General Microbiological Culture Collection Center (CGMCC, No. 0877, NCBI Assembly No. GCF_000187405.1) [25]. The WT strain and transformants generated in this study were grown on 1/4 SDAY (1% dextrose; 0.25% peptone; 0.5% yeast extract and 2% agar) and incubated for 15 days at 28 °C. The influence of nutrient on conidiation was determined on SYA medium (sucrose 3%, yeast extract 0.5%, NaNO_3_ 0.3%, K_2_HPO_4_ 0.1%, MgSO_4_·7H_2_O 0.05%, KCl 0.05%, FeSO_4_·7H_2_O 0.001%, MnSO_4_·H_2_O 0.001% and agar 1.8%) or SYA with replaced sucrose by other carbon source. Dextrose, agar, NaNO_3__,_ MgSO_4_·7H_2_O and FeSO_4_·7H_2_O were purchased from Chengdu Kelon Chemical Co., Ltd. (Chengdu, China); yeast extract and peptone were purchased from Beijing Auboxing Biotechnology Co., Ltd. (Beijing, China); sucrose was purchased from Sinopharm Chemical Reagent Co., Ltd. (Shanghai, China). All chemicals were purchased from Chongqing Chuandong Chemical Co., Ltd. (Chongqing, China) unless stated separately.

### 2.2. Deletion, Complementation and Overexpression of the Mavib-1 Gene

The *Mavib-1* (Gene ID: MT954969) disruption mutant was constructed by homologous recombination. For *Mavib-1* deletion, upstream and downstream flanking fragments (~1 kb) of *Mavib-1* coding sequences were amplified and inserted into the pK2-PB vector which harbored a *bar* cassette. The resulting vector pK2-PB-*Mavib-1*-LR was transformed into *M. acridum* mediated by *Agrobacterium tumefaciens* [26]. The *Mavib-1* disruption mutants (Δ*Mavib-1*) were screened on Czapek-Dox medium (sucrose 30 g/L, NaNO_3_ 2 g/L, KH_2_PO_4_ 1 g/L, MgSO_4_·7H_2_O 0.5 g/L, KCl 0.5 g/L, FeSO_4_·7H_2_O 0.01 g/L and agar 18 g/L) containing 500 μg/mL glufosinate-ammonium (Sigma, St. Louis, MO, USA). To rescue the deleted *Mavib-1*, the full-length *Mavib-1* sequence including flanking regions (5.0 kb in total length) was amplified and inserted into the pK2-*sur* vector containing the chlorimuron ethyl resistance gene *sur* [27] to construct the complementary vector pK2-*sur*-*Mavib-1*. This vector was transformed into Δ*Mavib-1* and the complementary transformants (CP) were screened on Czapek-Dox plate containing 60 µg/mL chlorimuron ethyl (Sigma, Bellefonte, PA, USA). To create an *Mavib-1* overexpression mutant, the *Mavib-1* gene was fused with *EGFP* and driven by the promotor of constitutive glyceraldehyde 3-phosphate dehydrogenase gene (*Magpd*). This vector was transformed into the WT and transformants (OE) were screened on Czapek-Dox plates supplemented with 500 µg/mL glufosinate-ammonium (Sigma, St. Louis, MO, USA). The transformants were verified by PCR, and florescence was observed under a laser scanning confocal microscope (LSCM, TCS SP8, Leica, Germany).

### 2.3. Transcription Activation Experiment

For the transcriptional activation test, full-length cDNA, N-terminal (contain NDT80_PhoG domain) and C-terminal of *Mavib-1* was amplified from a cDNA library with primer pairs *Mavib-1*-BD-F1/*Mavib-1*-BD-R2, *Mavib-1*-BD-F1/*Mavib-1*-BD-R1, *Mavib-1*-BD-F2/*Mavib-1*-BD-R2 (Table S1), respectively, and the amplified fragments were respectively inserted into the vector pBKT7 (Clontech) between the *Nde*I and *Eco*RI sites under the control of the GAL4 promoter to generate plasmid pBKT7-*Mavib-1* for transformation into the Yeast Y2H Gold strain. Putative prototrophic transformants were selected on a Trp-free SD medium and further transferred onto Trp/His/Ade-free SD plates containing 0.5 mM 5-Bromo-4-Chlroro-3-Indolyl-D-Galactopyranoside (X-α-gal, TaKaRa, Dalian, China) for transcriptional activation assay.

### 2.4. Stress Tolerance Assays

For the stress assays, aliquots of 2 µL conidial suspensions (1 × 10^6^ conidia/mL) of the WT, Δ*Mavib-1*, CP and OE strains were pipetted onto 1/4 SDAY plates supplemented with 1 mol/L NaCl, 0.01% SDS, 1 mol/L sorbitol (SOR) (Dingguo, Beijing, China), 500 µg/mL Congo Red (CR) (Solarbio, Beijing, China), 6 mmol/L H_2_O_2_, 50 µg/mL calcofluor white (CFW) (Solarbio, Beijing, China), respectively. The plates were incubated at 28 °C for 5 days. Stress tolerance to heat and ultraviolet radiation was assessed as described previously [4]. Half inhibition time of germination (IT_50_) under UV radiation or heat was then calculated for each strain. The assays were conducted with three replicates per treatment and the experiment was repeated three times.

### 2.5. Conidial Germination

Conidial germination assay was conducted on 1/4 SDAY plates as described previously [28]. The conidia of *M. acridum* WT, Δ*Mavib-1*, CP and OE strains were suspended in sterile water (1 × 10^7^/mL). Aliquots of 50 µL conidia suspension were spread on 1/4 SDAY plates and the plates were incubated at 28 °C. The conidial germination rate of each strain was determined every 2 h (cultured for 2, 4, 6, 8, 10 and 12 h, respectively) until conidia germinate almost completely. The half time to germination (GT_50_) was statistically calculated, and the data were analyzed. Three replicates in each independent experiment were conducted, and in each replicate, 300 conidia were used.

### 2.6. Electronic Microscopy

Ultrastructure of mature conidial cell wall surface of the WT, Δ*Mavib-1*, CP and OE strains was examined by scanning electronic microscopy (SEM, SU8010, Hitachi, Japan) as described previously [29]. Conidia of WT, Δ*Mavib-1*, CP and OE strains of *M. acridum* were collected, fixed with 2.5% glutaraldehyde solution (Servicebio, Wuhan, China) at 4 °C overnight, and then the samples were processed as follows: remove the fixation solution, rinse the samples three times with 0.1 M phosphate buffer, pH 7.0, 15 min each time; fix the sample with 1% osmic acid solution for 1–2 h; carefully remove the osmic acid waste solution, rinse the sample three times with 0.1 M, pH7.0 phosphate buffer for 15 min each time; use a gradient concentration (including 30%, 50%, 70%, 80%, 90% and 95%) ethanol solution to dehydrate the samples with each concentration for 15 min, and then treated with 100% ethanol twice, each time for 20 min. Rinse the sample with a mixture of ethanol and isoamyl acetate (*v/v* = 1/1) for 30 min, and then rinse with pure isoamyl acetate for 1h or leave the samples overnight. After critical point drying and coating, the samples were observed in a scanning electron microscope.

### 2.7. Conidial Hydrophobicity Assay

Conidial hydrophobicity was determined with hexadecane as described previously [29]. Conidia of WT, Δ*Mavib-1*, CP and OE strains were suspended (3 × 10^7^ conidia/mL) in reaction buffer (22.2 g/L K_2_HPO_4_, 7.26 g/L KH_2_PO_4_, 1.8 g/L urea, 0.2 g/L MgSO_4_, pH 7.1). Aliquots of 1 mL conidia suspension (OD_470_ = 0.4) were mixed well with 100 μL hexadecane and then incubated at 25 °C for 10 min. The upper hexadecane was discarded. The absorption peak of conidia suspension at 470 nm (A_470_) was measured after removing solidified hexadecane completely after incubation at 4 °C for 30 min. The hydrophobic index was calculated using the following formula: (A_470_, control-A_470_, hexadecane-treated)/(A_470_, control). The assays were conducted with three replicates per treatment and the experiment was repeated three times.

### 2.8. Conidiation and Colony Morphology with Various Carbon or Nitrogen Sources

SYA containing different carbon sources at 3% (*w/v*) (glucose, sucrose and glycerol, respectively) or SYA containing different nitrogen sources at 3% (*w/v*) (urea, glutamine/Gln, NaNO_3_, respectively) were used for colony growth assays and conidiation analysis. For conidiation observation under microscope, each plate was spread evenly with 100 µL of conidial suspension (1 × 10^7^ conidia/mL) and incubated at 28 °C for 12–36 h. For colony growth assay, aliquots of 2 µL of conidial suspension (1 × 10^6^ conidia/mL) of each strain were inoculated on different media and the plates were incubated at 28 °C for 5 days. For conidial yield assay, 2 µL conidial suspensions (1 × 10^6^ conidia/mL) of WT, Δ*Mavib-1*, CP and OE were spotted onto ¼ SDAY medium in a 24-hole plate and incubated at 28 °C. Conidia were collected after growing for 15 days and vortexed with 1 mL of 0.05% Tween 80. Conidial concentration was determined using a hemocytometer under a microscope. All experiments were performed three times.

### 2.9. RNA-Seq

Transcriptional profiles were measured in WT, Δ*mavib-1* and OE strains by RNA-Seq. Total RNA was extracted from WT, Δ*mavib-1*, or OE strains after growing on SYA plates at 28 °C for 20 h. Total RNA was extracted from each sample using an RNA Kit (Invitrogen, Carlsbad, CA, USA) with Rnase-free DnaseI added and reverse transcribed into cDNA with an oligo-dT primer using the PrimeScript RT Master Mix (TaKaRa, Dalian, China). Sequencing libraries were prepared, and sequencing was conducted on BGISEQ-500 (BGI, Beijing, China). Genes were classed as significantly differentially expressed when false discovery rate (FDR) <0.001 and fold change ≥2.

Differentially expressed genes (DEGs) were classified and annotated using gene ontology (GO) and Kyoto Encyclopedia of Genes and Genomes (KEGG) pathway analysis.

### 2.10. Reverse Transcription Quantitative PCR (RT-qPCR)

The gene expression levels were examined by RT-qPCR. Amplification mixtures (20 µL) contained 1 µL template cDNA, 10 µL IQ SYBR Green Supermix (2×, Bio-Rad, Foster City, CA, USA) and 1 µL of each primer (qF/qR, 10 µmol/L) [27]. The reactions were performed in iCycler system (Bio-Rad). The *Magpd* gene (EFY84384), amplified with primers gpd-F/gpd-R (Table S1), was used as the endogenous control, as described previously [27]. Transcript ratios of the target gene were evaluated using the 2^−ΔΔCT^ method [30]. All PCR amplifications were conducted in triplicate, and trials were repeated three times.

### 2.11. Data Analysis

All measurements were analyzed using a one-way ANOVA model with the SPSS 19.0 program (SPSS Inc., Chicago, IL, USA). Tukey’s honestly significant difference test was used to evaluate means at α = 0.05.

## 3. Results

### 3.1. Structural Features and Deletion of Mavib-1

*Mavib-1* gene has a full-length sequence of 1991 bp containing two introns (67 bp and 79 bp). A cDNA sequence of 1845 bp (Acc. MT954969) encodes a protein of 614 amino acids with a molecular weight of 66.90 kDa and an isoelectric point of 6.84. The Mavib-1 protein contains an NDT80_PhoG domain with 165 amino acids (Residues 171–335) (Figure S1A), which is highly conserved in Vib-1 homologs among different fungi (Figure S1B). Phylogenetic analysis revealed that *Mavib-1* was clustered with other fungal NDT80_PhoG domain containing proteins (Figure S1C). To elucidate the functions of *Mavib-1* in *M. acridum*, Δ*Mavib-1*, CP and OE strains were constructed (Figure S2A), and the expected recombinant events were verified by PCR (Figure S2B), Semi-quantitative RT-PCR (Figure S2C) and RT-qPCR analysis (Figure S2D).

### 3.2. C-Terminal of Mavib-1 Has Transactivation Activity

**Figure 1:**
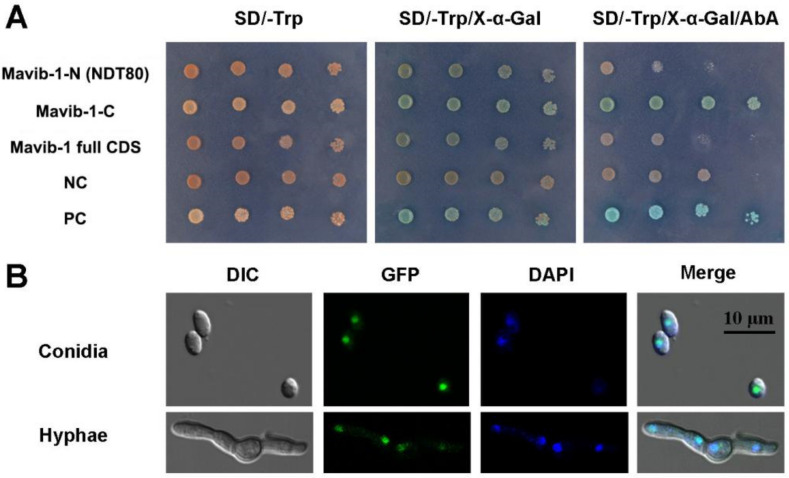
Transcriptional-activation assays and subcellular localization of Mavib-1. (**A**) Transcriptional activation assays in yeast. Mavib-1-N (containing NDT80 domain), Mavib-1-C or Mavib-1 full CDS were fused to the GAL4 DNA-binding domain and expressed in yeast strain Y2H Gold. Strain containing only GAL4 DNA-binding domain was as negative control (NC), containing the GAL4 DNA-binding and activation domains were as positive controls (PC). All the yeast strains were cultured on SD-Trp plates, SD-Trp plates containing 0.5 mM X-α-gal and SD-Trp plates containing 0.5 mM X-α-gal and 200 ng/mL AbA (Aureobasidin A) at 30 °C for 3 days. (**B**) Subcellular localization of Mavib-1. The expression of *Mavib-1* and *EGFP* fusion protein was driven by strong promoter *PgpdM*. The conidia and mycelia of OE strain were stained with DAPI (4′,6-diamidino-2-phenylindole, C0065, Solarbio, China), a fluorescent dye that can bind strongly to DNA, and observed under a confocal microscope (TCS SP8, Leica, Germany).

Mavib-1-N (containing NDT80 domain), Mavib-1-C or Mavib-1 full CDS were fused to the GAL4 DNA-binding domain and expressed in yeast strain Y2H Gold. Mavib-1-C fusion strain showed blue spots on SD-Trp plates containing X-α-gal and AbA, indicating a transactivation activity at C-terminal of Mavib-1 in yeast (Figure 1A). To investigate the subcellular localization of Mavib-1, *Mavib-EGFP* was expressed in *M. acridum*. Results showed that green fluorescent signal was observed in the nucleus in both conidia and mycelia and overlapped with the staining of DAPI dye, indicating a nuclear localization of Mavib-1 (Figure 1B). Therefore, consistent with *Vib-1* in *A. nidulans* and *N. crassa* [22,23], *Mavib-1* can functionally act as a transcription factor in *M. acridum.*

### 3.3. Mavib-1 Affected Stress Tolerances of M. acridum

**Figure 2:**
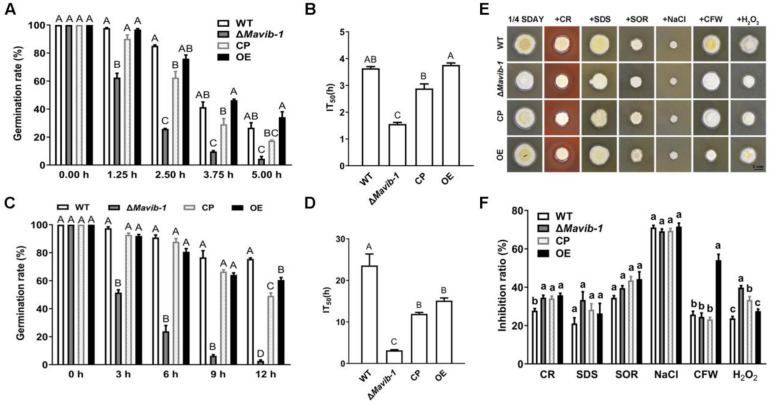
Stress tolerances of conidia from WT, Δ*Mavib-1*, CP and OE strains. (**A**) Germination rate of each strain on 1/4 SDAY media at 28 °C for 20 h after UV-B stress treatment at 0, 1.25, 2.5, 3.75, 5.0 h. (**B**) The half inactivity time (IT_50_) of each strain with UV-B treatment. (**C**) Germination rates on 1/4 SDAY at 28 °C for 20 h after 46 °C heat stress treatment at 0, 3, 6, 9, 12 h. (**D**) The IT_50_ of WT, Δ*Mavib-1*, CP and OE strains under 46 °C heat treatment. (**E**) The fungal colony on 1/4 SDAY medium and 1/4 SDAY with cell wall disruptors (500 µg/mL CR, 50 µg/mL CFW), cell wall stressor (0.01% SDS), hyperosmotic stressors (1 mol/L SOR or 1 mol/L NaCl), oxidative stress (6 mmol/L H_2_O_2__)_, respectively. (**F**) Inhibition rate of colony growth. All experiments were repeated three times for statistical analysis. Different capital letters represented significant difference at *p* < 0.01; different lower-case letters represented significant difference at *p* < 0.05.

Stress tolerances are very important characteristics for fungal potential against insect pests. Compared to the WT, the Δ*Mavib-1* mutant showed a significant change in conidial sensitivity to UV radiation and heat stress (Figure 2). After UV-B irradiation, conidial germination and mean IT_50_ values of Δ*Mavib-1* mutant were significantly decreased compared to WT, while OE strain did not show significant difference compared to WT (Figure 2A,B). After heat treatment, both Δ*Mavib-1* and OE strains presented a significant decrease in conidial germination and mean IT_50_ values compared to WT, but Δ*Mavib-1* showed a sharply decreased in heat treatment (Figure 2C,D). CP strain showed a partial recovery in UV-B and heat stress tolerance compared to WT and Δ*Mavib-1* mutant.

Δ*Mavib-1* and OE did not show significant difference in tolerance to SDS, SOR and NaCl compared to WT. The OE strain had more sensitivity to CR and CFW, while Δ*Mavib-1* had weaker tolerance to CR and H_2_O_2_ (Figure 2E,F), indicating a destroyed cell wall integrity in Δ*Mavib-1* and OE strains.

### 3.4. Disruption of Mavib-1 Affected Conidial Germination, Conidial Cell Wall Surface Ultrastructure, and Conidial Hydrophobicity

**Figure 3:**
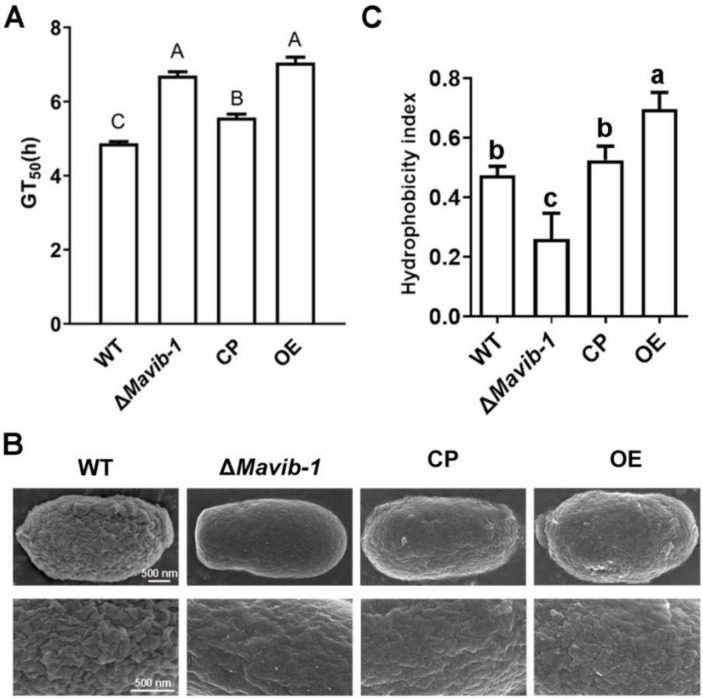
Conidial germination, conidial surface morphology and hydrophobicity determination. (**A**) The 50% germination times (GT_50_) of WT, Δ*Mavib-1*, CP and OE strains. (**B**) The conidial surface morphology by SEM. (**C**) Conidial hydrophobicity index. The trial was repeated three times. Different capital letters represented significant difference at *p* < 0.01 and different lower-case letters indicate significant difference, *p* < 0.05.

Germination rates of conidia of WT, Δ*Mavib-1*, CP and OE strains were determined on 1/4 SDAY at different culture intervals. Results showed that GT_50_ of Δ*Mavib-1* and OE strains were significantly prolonged than that of WT (Figure 3A). The *Mavib-1* gene affected the resistance to cell-wall-disrupting agent CFW (Figure 2E,F); we speculated that *Mavib-1* might affect the conidial cell wall structure. SEM analysis showed that the conidial surface of WT and OE strains had a rough cell wall surface, while the Δ*Mavib-1* strain had a smoother cell wall surface compared with WT (Figure 3B).

The structure of the fungal cell wall surface is closely related to the conidial characteristics. The rodlets on the conidial surface are arranged according to certain rules by fungal hydrophobins [31]. Hydrophobicity assay showed that the conidial hydrophobic index was significantly decreased in Δ*Mavib-1* and significantly increased in OE strain compared to WT (Figure 3C). These results indicated that deletion of *Mavib-1* affected conidial germination, conidial cell wall surface ultrastructure and conidial hydrophobicity in *M.* *acridum.*

### 3.5. Disruption of Mavib-1 Affected Growth and Conidiation of M. acridum on Different Carbon or Nitrogen Source Media

**Figure 4:**
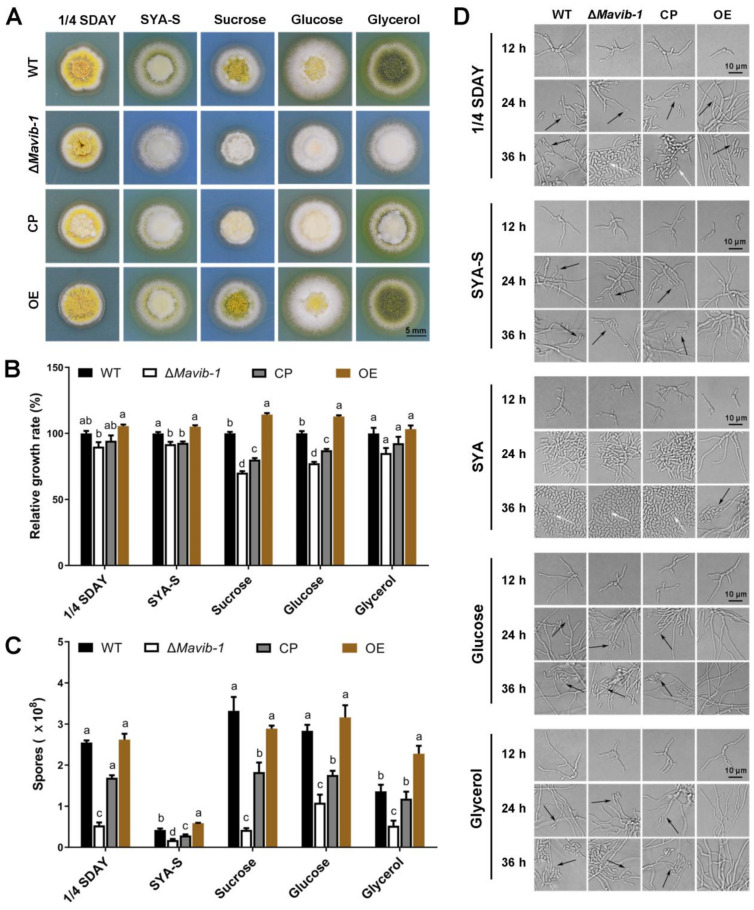
Colony growth and conidiation of fungal strains on media containing different carbon sources. (**A**) Colony morphology of WT, Δ*Mavib-1*, CP and OE strains. Fungal strains were cultured on the nutrient-rich medium 1/4 SDAY, and nutrient-limited medium SYA with easy-to-use or difficult-to-use carbon sources (3% *w/v*; easy to use: glucose; middle: sucrose, difficult to use: glycerol) for 5 d. Colony relative growth rate (**B**) and conidial yield (**C**) of WT, Δ*Mavib-1*, CP and OE strains cultured on 1/4 SDAY or SYA with different carbon sources for 5 d. The trial was repeated three times. Error bars are standard deviations of three replicates. Different lower-case letters indicate significant difference at *p* < 0.05. (**D**) Conidiation of WT, Δ*Mavib-1*, CP and OE strains on different media. The black arrows indicate normal conidiation, and the white arrows indicate microcycle conidiation.

*Mavib-1* has a nutrient sensing-related NDT80_PhoG domain, and the growth, conidiation and conidial yield of WT, Δ*Mavib-1*, CP and OE strains on media with different carbon and nitrogen sources were determined. On the nutrient-rich medium 1/4 SDAY, Δ*Mavib-1* and OE strains had similar colony color and size compared to WT. On nutrient-limited media, in which glucose, sucrose or glycerol was used as the main carbon source, respectively, or no extra carbon source was added, Δ*Mavib-1* colony had a much lighter color and significantly smaller size than WT, while the OE strain had a similar colony phenotype as WT. Conidial production analysis showed that deletion of *Mavib-1* resulted in decreased conidial yield of *M. acridum* regardless of rich nutrient or starvation (Figure 4C). These results indicated that *Mavib-1* gene is necessary for colony growth and the conidial melanin formation under nutrient deficiency in *M. acridum*. Microscopic observation showed that the Δ*Mavib-1* strain performed normal conidiation similar to WT and OE at the early growth time on 1/4 SDAY (12 h and 24 h). However, Δ*Mavib-1* strain produced more conidia than WT and OE at 36 h. Conidiation was also analyzed on SYA, a microcycle conidiation medium for *M. acridum* [4]. It clearly showed that WT and Δ*Mavib-1* conducted MC, while the OE strain changed to typical hyphal growth and conducted normal conidiation. On other media without a carbon source, or with glucose and glycerol as carbon source, Δ*Mavib-1* exhibited normal conidiation slightly earlier than WT, while the OE strain did not show conidiogenous structure until 36 h (Figure 4D).

**Figure 5:**
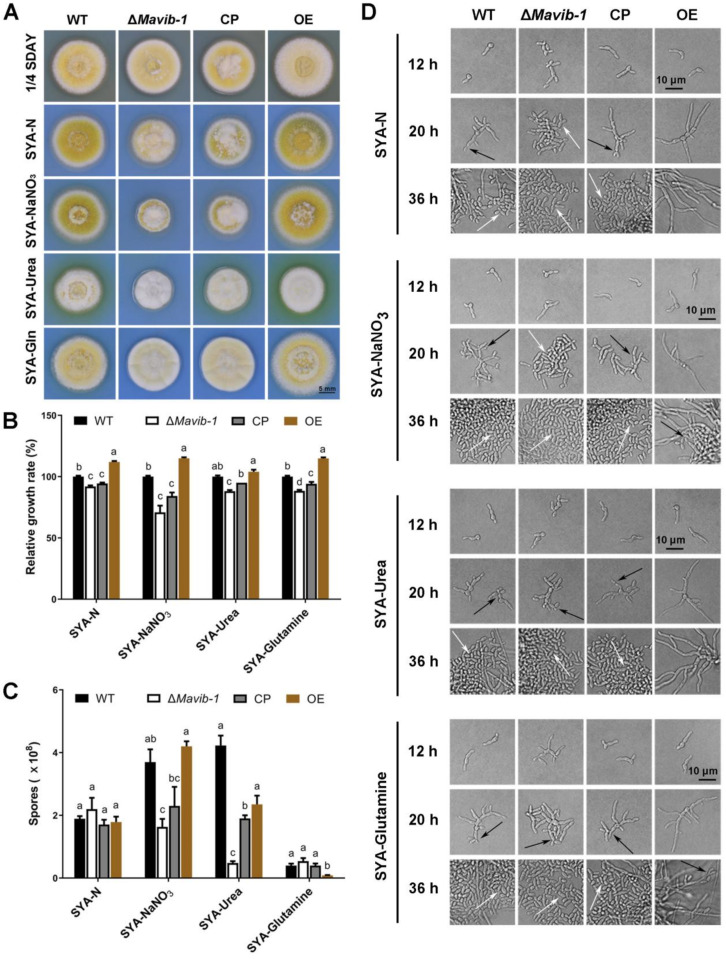
Colony growth and conidiation of Δ*Mavib-1*, CP and OE strains on media with different nitrogen sources. Colony morphology (**A**) and colony relative growth rate (**B**) of Δ*Mavib-1*, CP and OE strains on the media 1/4 SDAY, and SYA with different nitrogen sources. (**C**) Conidial yield of fungal strains cultured on different media for 15 d. Media contained 0.3% nitrogen source: SYA-N (SYA without NaNO_3_), urea (difficult to use), NaNO_3_ (middle), Gln (easy to use). Each trial was repeated three times for statistical analysis. Different lower-case letters mean significant difference at *p* < 0.05. (**D**) Conidiation of Δ*Mavib-1*, CP and OE strains on different media. The black arrows indicate NC, and the white arrows indicate MC.

Similar to the role in carbon source utilization, *Mavib-1* also contributed to the utilization of different nitrogen sources, including dominant (glutamine) and non-dominant (urea and NaNO_3_) nitrogen sources (Figure 5A). Δ*Mavib-1* colonies were whitish in color on SYA medium with different nitrogen sources (Figure 5A) and had significantly smaller size than WT and CP, when urea or NaNO_3_ was used as the main nitrogen source or no extra nitrogen source (SYA-N) was included, while the OE strain had an obviously larger colony on these media (Figure 5A,B). These results indicated that *Mavib-1* positively regulated fungal growth by affecting utilization of nitrogen sources. Conidial yield analysis showed that Δ*Mavib-1* had significantly decreased conidia production compared to WT when urea or NaNO_3_ was used as main nitrogen source, while overexpression of *Mavib-1* did not significantly affect conidial yield in *M. acridum* (Figure 5C). Microscopic observation showed that, similar as the results on the media with different carbon sources, Δ*Mavib-1* strain performed MC on SYA-N and SYA medium slightly earlier compared to WT. WT strain performed hyphal growth at 20 h, while Δ*Mavib-1* strain had already produced a mass of conidia at that time. Conidiation of OE strain was drastically delayed compared with WT on different nitrogen source media, and no conidia was formed until 36 h on the SYA-N and SYA-Urea media (Figure 5D). These results indicated that *Mavib-1* positively regulated fungal growth and negatively regulated conidiation of *M. acridum* by affecting utilization of different carbon or nitrogen sources especially when nutrient was insufficient. However, *Mavib-1* contributed to total conidial yield in *M. acridum* under both nutrient-rich and nutrient-limited conditions.

### 3.6. Mavib-1 Affected Expression of CAZyme Genes of M. acridum

To elucidate the mechanism by which *Mavib-1* affects the conidiation of *M. acridum* in response to different carbon and nitrogen sources, RNA-seq was performed for WT, Δ*Mavib-1* and OE strains cultured on SYA medium for 20 h (Accession No. PRJNA73660). The RNA-seq data were verification by RT-qPCR, and the difference fold changes obtained by the two methods were extremely significantly correlated (R = 0.890 and *p* < 0.01 in Δ*Mavib-1*, R = 0.960 and *p* < 0.01 in OE), indicating that the RNA-seq data were reliable (Table S2). Primer sequences used for verification are listed in Table S1.

**Figure 6:**
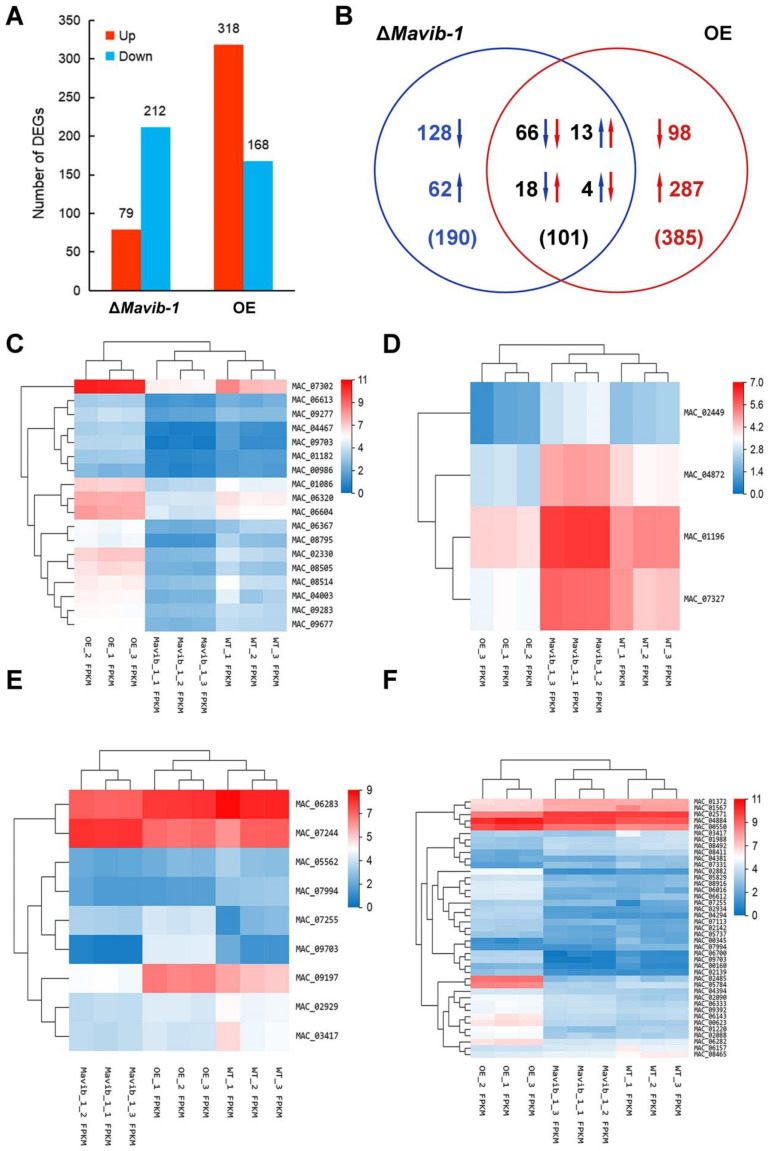
DEGs analysis. (**A**) Statistical histogram of the number of DEGs. (**B**) Venn diagram of DEGs. (**C**) Heat map of 18 DEGs downregulated in Δ*Mavib-1* and upregulated in OE. The 18 DEGs consistent with the change trend of *Mavib-1* were analyzed by cluster analysis of expression patterns. The distance calculation method was used: the spearman correlation coefficient between samples, the Pearson correlation coefficient between genes. (**D**) Cluster heat map of the expression levels of the 4 genes upregulated in Δ*Mavib-1* and downregulated in OE. (**E**) Heat map of the expression of DEGs in Δ*Mavib-1* annotated as CAZyme genes. (**F**) Heat map of the expression of DEGs in OE annotated as CAZyme genes. The up and down arrows represent upregulated or downregulated genes, respectively. Numbers in blue represent Δ*Mavib-1* and red represent OE. The red color denotes the upregulated DEGs, and the blue color denotes downregulated DEGs. The log_2_ (expression value + 1) of the sample were shown in the horizontal axis, and DEGs are shown at the right-hand vertical side.

Statistical analysis was performed on the RNA-seq data of three groups. The correlation heat map showed that the correlation between the three replicates in each group was high, and the correlation between groups was small, indicating a good repeatability of sequence data (Figure S3A). Principal component analysis (PCA) showed that the three replicates in each group could be clustered together, again indicating that the data were reproducible (Figure S3B). The RNA-seq data were screened after quality control and qPCR validation and genes with |log2FC |≥ 1, Qvalue ≤ 0.01 and FPKM ≥5 were counted as DEGs (Tables S3 and S4). RNA-seq showed that there were a total of 291 DEGs (79 up-regulated, 212 down-regulated) in the Δ*Mavib-1* strain, and a total of 486 DEGs (318 up-regulated and 168 down-regulated) in the OE strain (Figure 6A) compared to WT. The Venn diagram showed that there were 101 shared DEGs between Δ*Mavib-1* and OE (66 DEGs both down-regulated, 13 DEGs both up-regulated, and 18 DEGs down-regulated in Δ*Mavib-1* and up-regulated in OE, 4 DEGs up-regulated in Δ*Mavib-1* and down-regulated in OE) (Figure 6B). The detailed information of 22 DEGs (18 downregulated and 4 upregulated in Δ*Mavib-1*), with opposite changing trend of transcription in Δ*Mavib-1* and OE, is listed in Table S5. To intuitively reflect these DEGs with the opposite changing trend, a cluster heat map of 22 DEGs was constructed (Figure 6C,D).

GO classification (Figure S4), KEGG enrichment (Figure S5) and carbohydrate activity enzymes (CAZymes) annotation analysis were performed for DEGs. The up-regulated genes in Δ*Mavib-1* strain were mainly enriched in RNA polymerase, nitrogen metabolism and atrazine degradation, etc., and the down-regulated genes were mainly enriched in neuroactive ligand–receptor interaction, protein digestion and absorption, pancreatic secretion, influenza A and glycerolipid metabolism, etc. (Figure S5A,B). The upregulated genes in the OE strain were mainly enriched in microbial metabolism in diverse environment, metabolic pathways, and beta-alanine metabolism, etc. The downregulated genes were mainly enriched in amino sugar and nucleotide sugar metabolism, phenylpropanoid biosynthesis, neuroactive ligand–receptor interaction, protein digestion and absorption and metabolic pathways, etc. (Figure S5C,D). These results indicated that *Mavib-1* affected the carbon and nitrogen source metabolic pathways of *M. acridum*. Aligning the DEGs with the CAZy database [32] revealed that many DEGs belong to CAZymes (9 of the 291 DEGs in the Δ*Mavib-1* strain listed in Table S6, and 41 of the 486 DEGs in the OE strain listed in Table S7). Cluster heatmap of DEGs belonging to CAZymes showed that the expression of CAZyme genes in Δ*Mavib-1* and OE strains have changed (Figure 6E,F), indicating that *Mavib-1* played an important role in regulation of the expression of CAZyme genes in *M. acridum*. At the same time, 21 nitrogen source metabolism-related genes (listed in Table S8) were also found in DEGs. The transcription changes of these nitrogen metabolism and conidiation related genes in Δ*Mavib-1* and OE strains may provide explanations for the effects of *Mavib-1* on nitrogen metabolism and conidiation in *M. acridum*.

## 4. Discussion

The growth and development of filamentous fungi require suitable conditions such as temperature, humidity, pH, and nutrients. Filamentous fungi can sense the nutrient conditions in the environment and regulate their development. In this study, we demonstrated the role of *Vib-1* in NC and MC by affecting the nutrition utilization in entomopathogenic fungus *M. acridum*.

Mavib-1 shares the NDT80_PhoG domain with Ndt80 of *S. cerevisiae* [33], Vib-1, Fsd-1 and NCU04729 of *N. crassa* [34,35], and XprG and NdtA of *A. nidulans* [36,37,38]. Mavib-1 was localized at nuclear in both conidia and hyphae, while Vib-1-GFP was found in nuclear in hyphae and cytoplasmically localized in conidiophores and immature conidia in *N. crassa* [21]. This might cause some divergent roles of *Vib-1* in different fungi. Proteins containing NDT80_PhoG domains had been reported in model fungi yeast and *N. crassa*. In *S. cerevisiae*, Ndt80 is a meiosis-specific transcription factor that regulates the expression of metaphase genes and also regulated by the pachytene checkpoint [39,40,41]. In filamentous fungi, sporogenesis is a procedure of producing a new cell through cell division by mitosis, which *Vib-1* might be involved in the regulation of conidiation. Consistent with our result, *Vib-1* was involved in the regulation of conidiation in *N. crassa* [21].

Our findings indicate that *Mavib-1* positively regulates fungal growth, and negatively regulates conidiation especially under nutrient deficient conditions. Consistent with the decreased hyphal extension in *Vib-1* disruption mutant in *N. crassa* [21], Δ*Mavib-1* had shortened hyphae when growing on nutrient-rich medium. However, conidiation was decreased in Δ*Mavib-1* and showed no changes when *Mavib-1* was upregulated, in contrast with no changes in *Vib-1* mutant and decreased conidiation in *Vib-1* overexpression strain in *N. crassa* [21]*,* indicating a divergent role of *Vib-1* in different fungi. When under carbon-limited conditions, deletion of *N. crassa Vib-1* causes a growth defect on cellulose biomass [23]. Some other Ndt80-containing proteins were reported to contribute to growth and nutrient utilization. Ndt80 transcription factor RON1 is essential for hyphal growth and N-acetylglucosamine (GlcNAc) metabolism in *Crytococcus neoformans* [42]. In *A. nidulans*, the Ndt80-like protein XprG positively regulates the expression of extracellular proteases, mycotoxins and penicillins in response to carbon starvation [37]. In *M. acridum*, disruption of the *Vib-1* led to a more serious defect in growth and conidiation initiation under carbon and nitrogen limited condition, but OE strain displayed a contrast phenotype with Δ*Mavib-1*, demonstrating more important roles of *Mavib-1* in both carbon and nitrogen utilization under nutrient limitation. Therefore, the explanation for the conidiation pattern change of *Mavib-1* mutants was as follows. When *Mavib-1* was impaired, *M. acridum* could not obtain enough nutrients to support hyphal growth and then the conidiation was induced, while hyphal growth was retained and strengthened owing to an improvement of nutrient utilization during overexpression of *Mavib-1.*

Consistent with the inhibited hyphal growth in Δ*Mavib-1* and promoted growth in OE strain on plate, RNA-Seq analysis revealed that DEGs of Δ*Mavib-1* and OE strains included many CAZyme members, which can degrade, modify or create glycosidic bonds. In Δ*Mavib-1*, the DEGs related to CAZyme were mostly downregulted (seven downregulated in nine DEGs) compared to upregulation of most CAZyme DEGs (27 upregulated in 41 DEGs) in OE strain. This indicated a more active carbon metabolism when *Mavib-1* was overexpressed. Similar in *N. crassa, Vib-1* can repress glucose signaling and CCR [23], and cellulose digestion-related genes were increased and glucose metabolism related CAZyme were repressed under carbon starvation [22,23] in *Vib-1* disruption mutant.

In *Aspergillus niger*, nutrient limitation, such as severe carbon limitation, led to a zero growth rate and induction of conidiation [43,44]. *M. acridum* show similar growth phenotype on minimal medium (Figure S6A), on which all WT and mutant strains conducted MC and no hyphal growth was observed under microscope (Figure S6B). This made it impossible to analyze the shift between hyphal growth and conidiation. On minimal medium, OE strain had a larger colony size and a better growth than Δ*Mavib-1*, which was consistent with the result on nutrient-limited medium SYA (Figure S6A). Therefore, we preferred analysis of hyphal growth and conidiation on nutrient-limited media SYA, on which *M. acridum* might change to normal conidiation when conidiation was in dysregulation.

The fungal cell wall acts as an environmental barrier to the host immune system and plays a crucial role in the pathogenic process and stress tolerance of entomopathogenic fungi [45]. Elements involved in pathways of cell wall integrity would affect the fungal virulence and stress tolerance to heat, UV irradiation or chemical stressors [46,47,48]. In *M. acridum*, *Vib-1* disruption or overexpression affected many genes related to CAZyme, which would also affect the cell wall synthesis. The conidial pigment formation-related gene laccase, also a virulence factor [49], was downregulated in Δ*Mavib-1* and upregulated in OE strain (Table S5), which was consistent with the light color of colony and decreased pathogenicity in Δ*Mavib-1*. Agreed with decreased UV tolerance, riboflavine-aldehyde-forming enzyme gene (MAC_01768), contributed to UV tolerance and virulence in *Metarhizium* [50], was significantly downregulated in Δ*Mavib-1*. SEM showed that Δ*Mavib-1* had abnormal conidial surface morphology compared to WT and OE. These changes in gene transcription or cell wall structure might lead to changes tolerance to environmental stresses and virulence. Δ*Mavib-1* did not show opposite transcription changes with *Mavib-1*-OE strain in all the tested phenotype, such as decreased virulence, decreased UV and heat tolerance in Δ*Mavib-1,* while there were no changes for these phenotypes in OE. This may be due to the complexity of biological regulation. After a gene is knocked out or overexpressed, it may lead to expression changes of other related genes to make up for the defects, and finally result in an atypical phenotype. Previous reports showed that the damage of conidial cell wall surface results in reduced pathogenicity, but enhanced thermotolerance in *B. bassiana* [31]. Moreover, conidial characteristics are closely related to the adherence and invasion ability of entomopathogenic fungi [29,51]. The changes in conidial characteristics in Δ*Mavib-1* would possibly affect the fungal pathogenicity. Some pathogenicity-related genes were also affected by *Mavib-1*. For example, citrinin biosynthesis transcriptional activator CtnR [52] and polyketide synthase protein (MAC_01101) [53], were differentially expressed in Δ*Mavib-1* or OE strains (Tables S3 and S4). These results suggested that *Vib-1* might contribute to virulence in *M. acridum*.

## 5. Conclusions

In summary, our study indicated that the transcription factor Mavib-1 positively regulated fungal growth and negatively regulated conidiation by affecting utilization of carbon and nitrogen sources in *M. acridum*. Owing to the contribution in the regulation of nutrient utilization, *Mavib-1* contributed to the conidiation pattern shift from microcycle conidiation to normal conidiation under nutrient deficiency. Moreover, *Mavib-1* is important for stress tolerance in *M. acridum*. Further analysis of the divergent upstream and downstream targets of Mavib-1 in regulating fungal growth, conidiation, stress tolerance and virulence will help explore the underlying regulative mechanisms of *Vib-1* in response to nutrient and other environmental stimuli.

## Data Availability

The data presented in this study are available in this article and its Supplementary Materials.

## Supplementary Materials

The following supporting information can be downloaded at: https://www.mdpi.com/article/10.3390/jof8060594/s1, Figure S1: Sequence analysis of Mavib-1. Figure S2: Construction of *Mavib-1* disruption (Δ*Mavib-1*), complementation (CP) and overexpression (OE) strains of the *Mavib-1* gene in *M. acridu**m*. Figure S3: RNA-seq data quality control analysis. Figure S4: GO classification of DEGs. Figure S5: DEGs KEGG pathway enrichment analysis. Figure S6: Colony growth and conidiation of WT, Δ*Mavib-1*, CP and OE strains on minimal medium (CZA). Table S1: Primers used in construction of *Mavib-1* deletion mutant, complemented strain and overexpression. Table S2: Verification of DGEs results by RT-qPCR analysis. Table S3: DEGs of Δ*Mavib-1* compared to WT cultured on SYA medium for 20 h. Table S4: DEGs of *Mavib-1* OE compared to WT cultured on SYA medium for 20 h. Table S5: DEGs with opposite changing trends of transcription in Δ*Mavib-1* and OE. Table S6 CAZyme in DEGs of Δ*mavib-1*. Table S7: CAZyme in DEGs of OE. Table S8: DEGs related to nitrogen metabolism.
