# Supplementary material for: Transcription Factor Mavib-1 Negatively Regulates Conidiation by Affecting Utilization of Carbon and Nitrogen Source in Metarhizium acridum
Source: J Fungi (Basel). 2022 Jun 1;8(6):594. doi: 10.3390/jof8060594 (PMC9224900; doi:10.3390/jof8060594)
Supplement: Supplementary file 1 [file jof-08-00594-s001.zip › jof-1728880-supplementary.pdf]

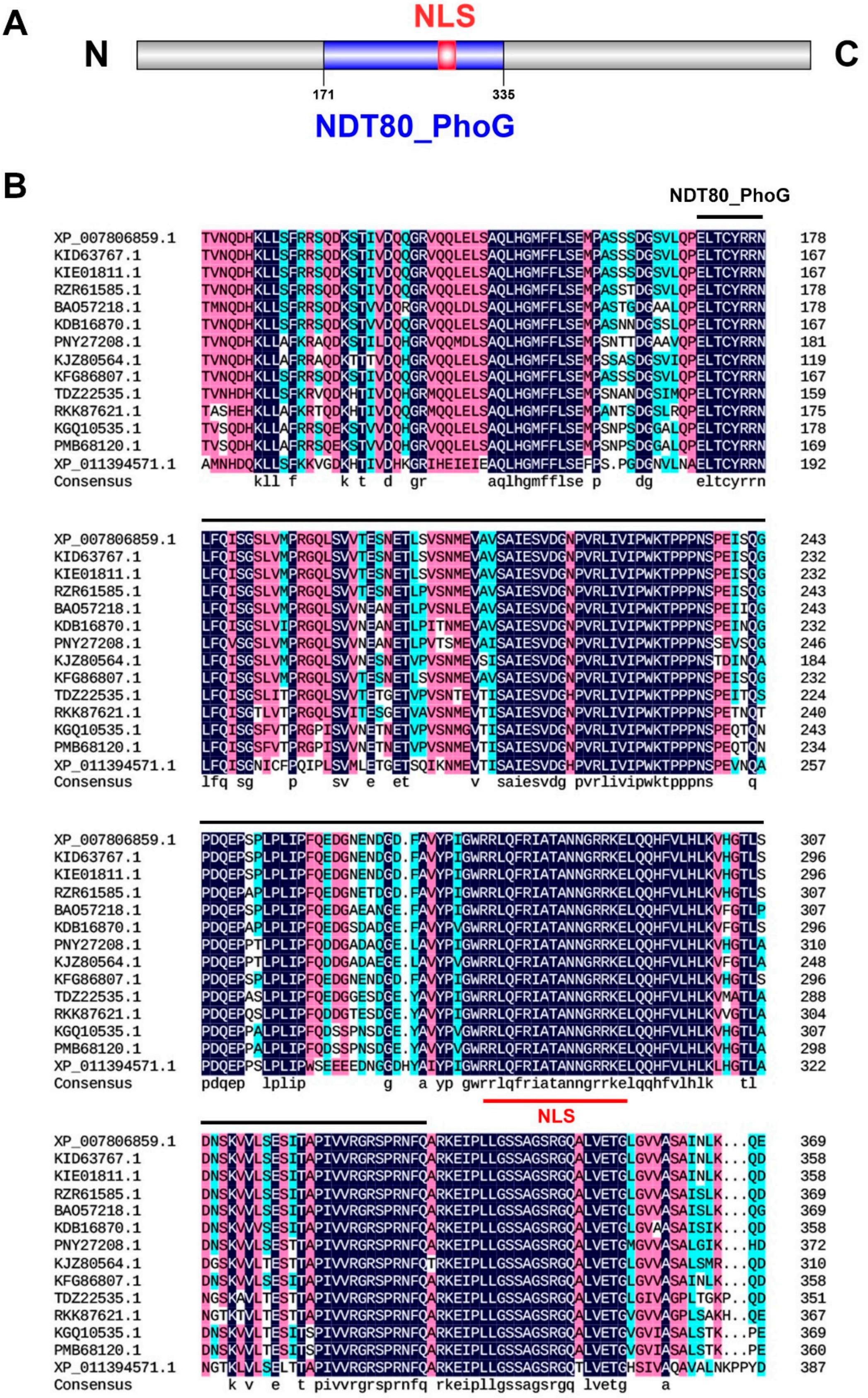
A
N
NLS
C
171
335
NDT80_PhoG
B
NDT80_PhoG
NLS
XP_007806859.1
KID63767.1
KIE01811.1
RZR61585.1
BAO57218.1
KDB16870.1
PNY27208.1
KJZ80564.1
KFG86807.1
TDZ22535.1
RKK87621.1
KGQ10535.1
PMB68120.1
XP_011394571.1
Consensus

C

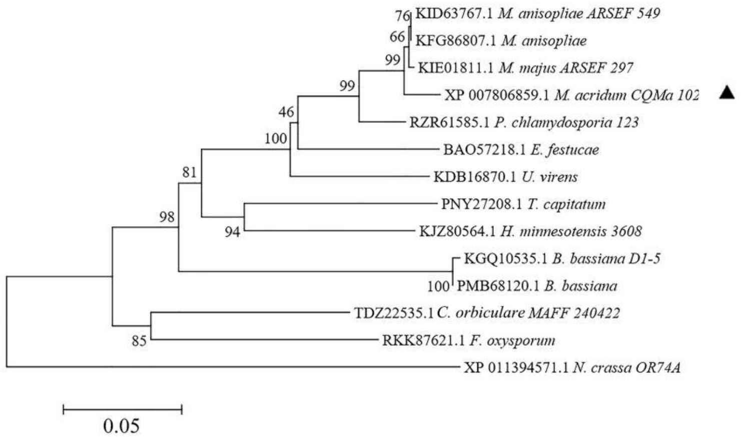

Figure S1 Sequence analysis of Mavib-1

A. Domain analysis of Mavib-1 by BLASTP. B. Sequence alignment of the NDT80_PhoG domain-containing protein sequences with DNAMAN. C. Phylogenetic analysis of Mavib-1 and its homologous proteins. The protein sequences were aligned with Clustal X and a neighbor joining tree was generated with 1,000 boot strap replicates using the program MEGA v6.0. Numbers at nodes represent the support value of homologs. Branch of Mavib-1 was indicated with black triangle. A phylogenetic tree was generated using MEGA v6.0 software using the neighbor joining method after the protein sequences were aligned by Clustal X, and it passed 1,000 boot strap tests. The phylogenetic tree based on vib-1 sequences from *M. acridum* (MT954969), *M. anisopliae ARSEF 549* (KID63767.1), *M. anisopliae* (KFG86807.1), *M.majus ARSEF 297* (KIE01811.1), *P. chlamydosporia 123* (RZR61585.1), *E. festucae* (GenBank: BAO57218.1), *U. virens* (KDB16870.1), *T. capitatum* (PNY27208.1), *H. mimesotensis 3608* (KJZ80564.1), *B. bassiana D1-5* (KGQ10535.1), *B. bassiana* (PMB68120.1), *C.orbiculare MAFF 240422* (TDZ2253.1), *F. oxysporum* (RKK87621.1), and *Neurospora crassa OR74A* (XP_011394571.1).

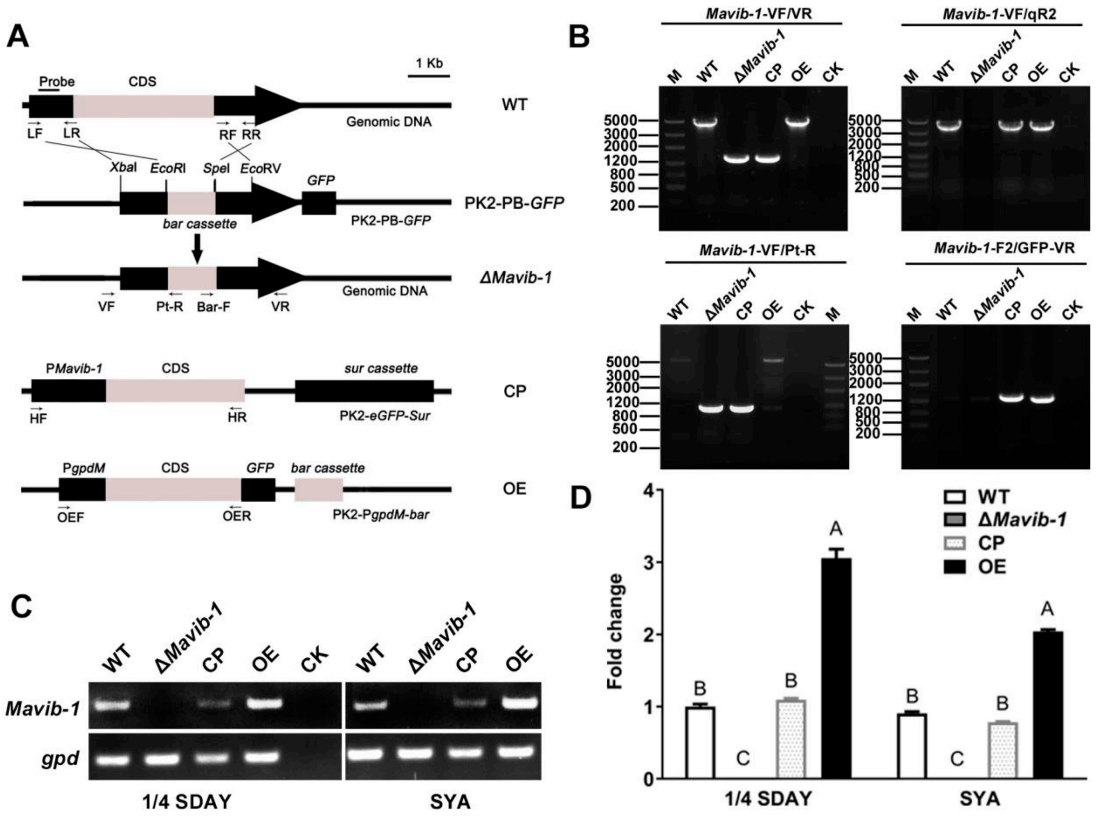

Figure S2 Construction of *Mavib-1* disruption (Δ*Mavib-1*), complementation (CP) and overexpression (OE) strains of the *Mavib-1* gene in *M. acridum*

A. Sketch map of the *Mavib-1* disruption, complementation and overexpression strains construction. Disruption vector pK2-pb-*GFP*-*Mavib*-1-LR with *bar* as a screening marker was transformed into WT. P*Mavib-1*-*Mavib-1*-*sur* was transformed into Δ*Mavib-1* to constructed CP strain and *PgpdM*-*Mavib-1*-*EGFP*-*bar* was transformed into WT to constructed OE strain. B. Verification of each genetic engineering strain by PCR amplification. Primer pairs *Mavib-1*-VF/*Mavib-1*-VR, *Mavib-1*-VF/*Mavib-1*-qR2, *Mavib-1*-VF/Pt-R and *Mavib-1*-F2/GFP-VR were used to screen transformants by PCR. C. RT-PCR analysis of *Mavib-1* in engineering fungal strains. D. RT-qPCR analysis of *Mavib-1* in engineering fungal strains, and the *gpd* gene as a reference.

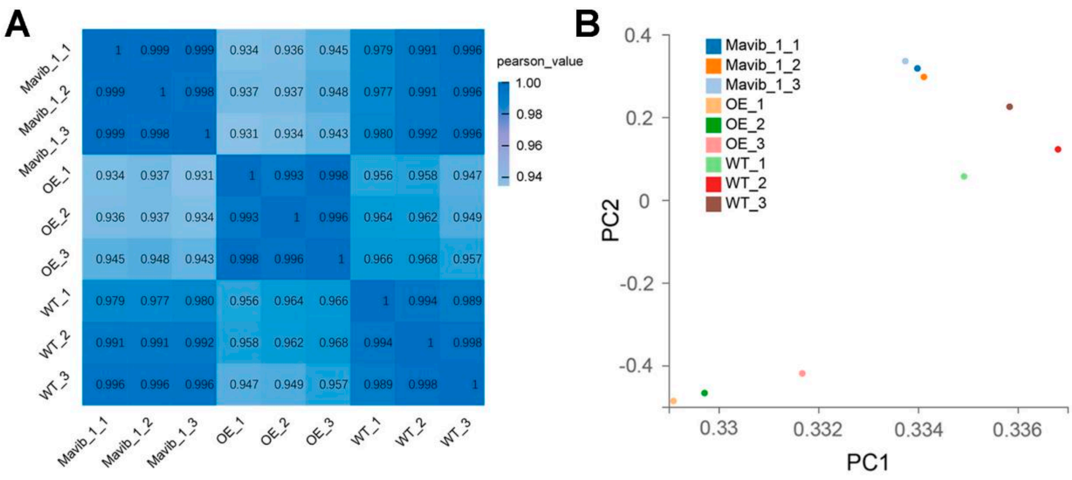

Figure S3 RNA-seq data quality control analysis

A. Correlation heat map. B. Principal component analysis (PCA chart).

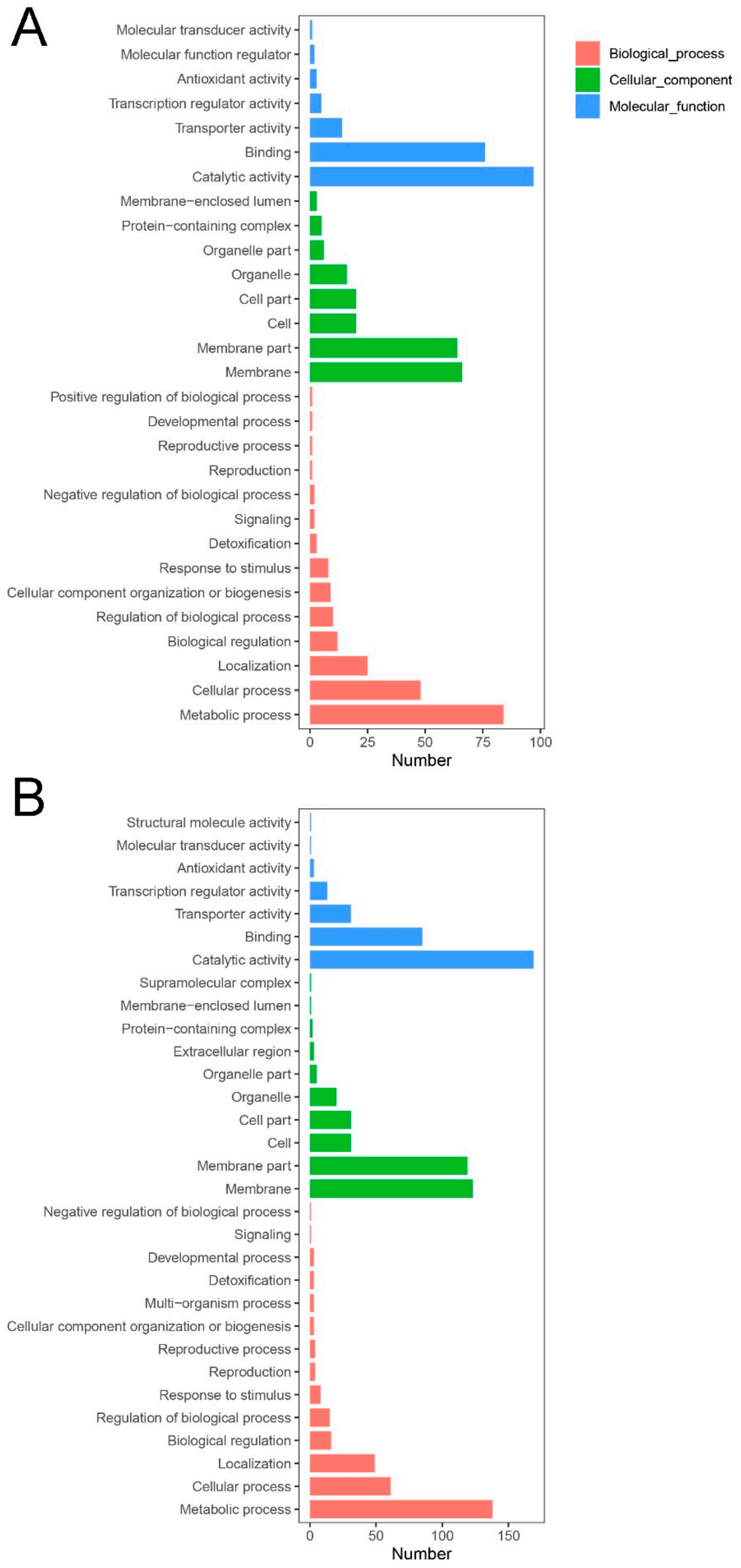

Figure S4 GO classification of DEGs

A. GO classification of DEGs in Δ*Mavib-1*. B. GO classification of DEGs in OE.

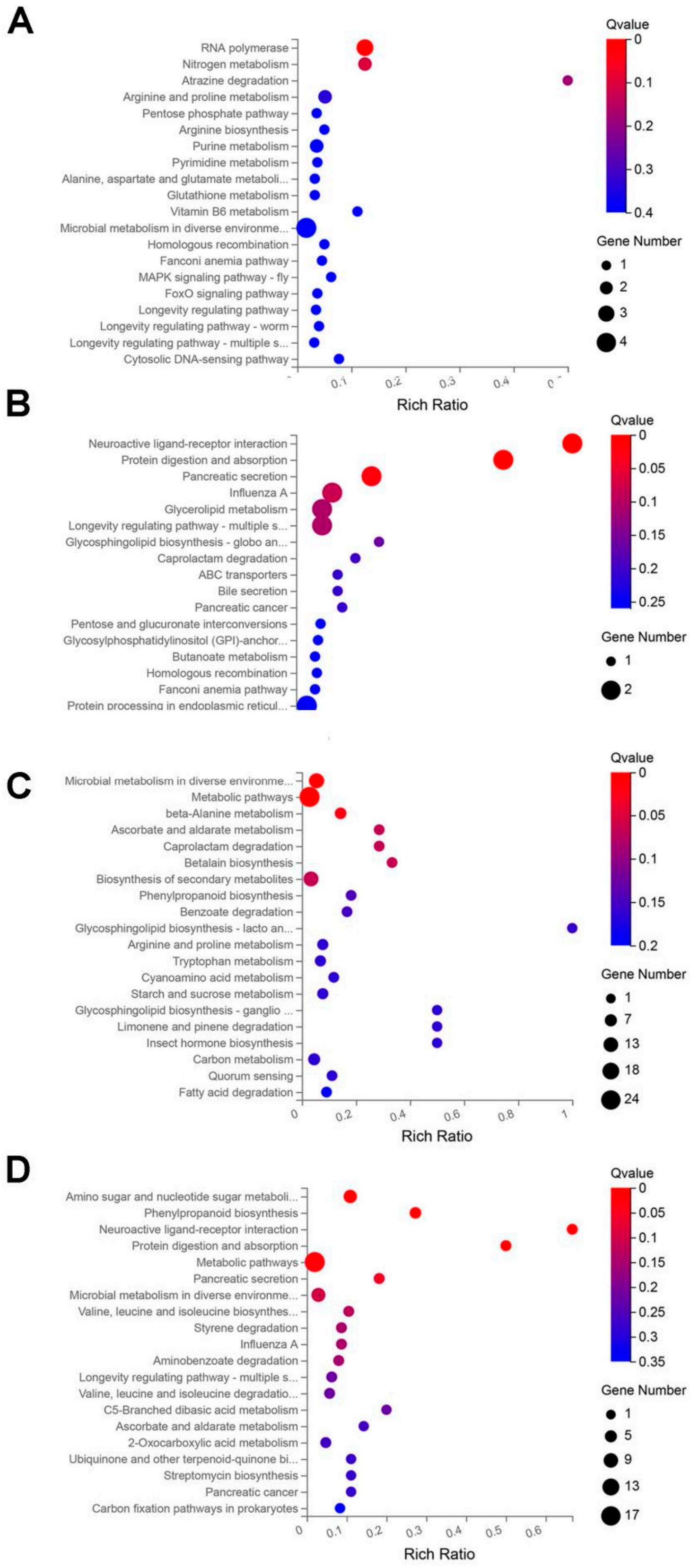

Figure S5 DEGs KEGG pathway enrichment analysis

A. Up-regulated genes in Δ*Mavib-1*; B. Down-regulated genes in Δ*Mavib-1*; C. Up-regulated

genes in OE; D. Down-regulated genes in OE. The X-axis is the enrichment ratio, the Y-axis is KEGG Pathway. The bubble size represents the number of genes annotated to a KEGG Pathway, and the color indicates the enriched Q-value. The darker color, the smaller Q-value.

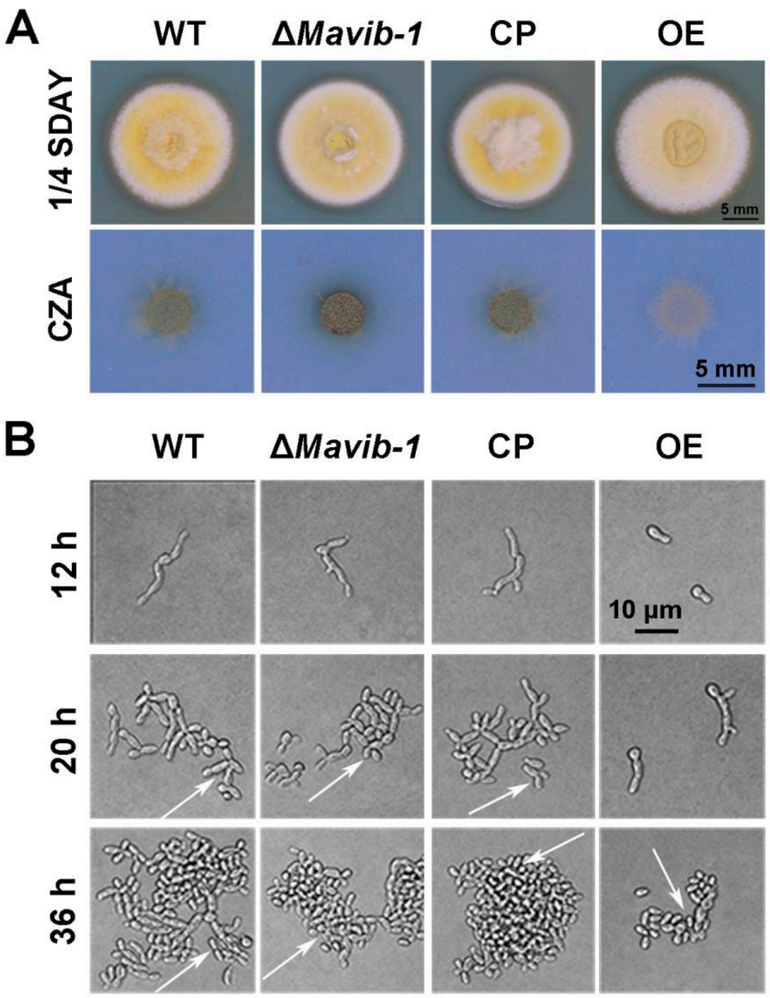

Figure S6 Colony growth and conidiation of fungal strains on minimal medium (CZA)

A. Colony morphology of Δ*Mavib-1*, CP, and OE strains. Fungal strains were cultured on the media 1/4 SDAY and CZA for 6 d. B. Conidiation of Δ*Mavib-1*, CP and OE strains on CZA medium. The white arrow indicates microcycle conidiation.

Table S1 Primers used in construction of *Mavib-1* deletion mutant, complemented and overexpression strains.

Primer name	Sequence (5'-3')	Note
**For cDNA amplification**		
Mavib-1-cF1	TGAACTAATATTTGCCTCCCACGGG	First PCR run
Mavib-1-cR1	GCCTACCGTTGTTCAGAGCATCTTG	
Mavib-1-cF2	ATGTCCCCATCCCACGGTAGCTCTT	Second PCR run
Mavib-1-cR2	AGCAGCTGTCCACGAGTAGTTGTTC	
**For mutant strains**		
Mavib-1-LF	GACTAGTGCAGAGGAGGAGAATAGCC	Cloning 5' homology arm
Mavib-1-LR	CGGAATTCAATACCACCAGGAGAAAGAAGT	
Mavib-1-RF	GCTCTAGACAGGCGAGACCCACTACTT	Cloning 3' homology arm
Mavib-1-RR	ACGATATCCCCTCATCTTCACGACCAC	
Mavib-1-VF	GCGATGCTTGCTCAACAG	Verification
Mavib-1-VR	CGTACTGAAGAGGCGGAAGT	*Mavib-1*-VF
Pt-R	CAGCCAAGCCCAAAAAGTG	Verification
Bar-F	GCTCTACACCCACCTGCT	Verification
**For CP strains**		
Mavib-1-HF	GACGGCCAGTGCCAAGCTGATGCTCGGTTCTACTCACAA	Cloning *Mavib-1*
Mavib-1-HR	CCTTGCTCACCATGGATCCCTACTACTAAGCAGCTGTCCACGA	
Sur-VF	TCTGAGGCTTGATGAGACCAC	Verification
Sur-VR	TGTTACAGAGCACCTCGAACTAA	Verification
**For OE strains**		

Primer name	Sequence (5'-3')	Note
Mavib-1-OEF	TACACACACGCAAATCTAGAATGACCGAG CTCCGAGGAGA	Cloning *Mavib-1*
Mavib-1-OER	CTCACCATACTAGTCTCGAGAGCAGCTGT CCACGAGTAGT	
Mavib-1-F2	TCAAGCCCGCAAAGAAAT	Verification
GFP-VR	CGATGCGGTTCACCAGGGTGT	Verification
**For transcription activation assay**		
Mavib-1-BD-F1	CTCAGAGGAGGACCTGCATATGATGATGA CTACGGCAGCATA	Cloning 5'-region of *Mavib-1*
Mavib-1-BD-R1	CGACGGATCCCCGGGAATTCGCGGGCTTG AAAATTCCGTG	
Mavib-1-BD-F2	CTCAGAGGAGGACCTGCATATGAAAGAAA TTCCTCTTCTTGGG	Cloning 3'-region of *Mavib-1*
Mavib-1-BD-R2	CGACGGATCCCCGGGAATTCAGCAGCTGT CCACGAGTAGT	
BD-F	TAATACGACTCACTATAGGG	Verification
BD-R	TAAGAGTCACTTTAAAATTTGTATAC	Verification
**For and yeast one-hybrid assay**		
Mavib-1-ADF	GTACCAGATTACGCTCATATGATGACCGAG CTCCGAGGAGA	Cloning CDS of *Mavib-1*
Mavib-1-ADR	ACGATTCATCTGCAGCTCGAGAGCAGCTG TCCACGAGTAGT	
P*vib*-pHIS2.1-YF	GACTCACTATAGGGCGAATTCGGGGCGAA CTCGTGATAC	Cloning promotor of *Mavib-1*
P*vib*-pHIS2.1-YR	ATAATGCCAGGAATTACTAGTGGGGCCAA GACATCATCATGG	

Primer name	Sequence (5'-3')	Note
AD-F	TAATACGACTCACTATAGGG	Verification
AD-R	AGATGGTGCACGATGCACAG	Verification
**For RT-qPCR**		
gpd-qF	GACTGCCCGCATTGAGAAG	149 bp
gpd-qR	AGATGGAGGAGTTGGTGTTG	
Mavib-1-qF2	GTAGGTGATGTCTCGCCAGG	228 bp
Mavib-1-qR2	AGCAGCTGTCCACGAGTAGTT	
MAC_08063-qF	CTTCAGACGAACCAGATT	154 bp
MAC_08063-qR	TACCTTTCCGAGATTCCT	
MAC_02470-qF	AATATAAGCACTCGTCCTAC	176 bp
MAC_02470-qR	TCTGACATTGGTTGACTG	
MAC_00601-qF	GTATTGCTCCGAGTTCTT	143 bp
MAC_00601-qR	CCAAGTCTTCCAGATTCC	
MAC_04059-qF	GACTGATTGACCTGTTCT	81 bp
MAC_04059-qR	CATTCTCCGTGCTCTTAT	
MAC_09082-qF	AGGTTCCAAGGCAGTATA	190 bp
MAC_09082-qR	GCGTAGATAGGTGATTAGTT	
MAC_00973-qF	GAGTGGATTGAGAGTTATGA	176 bp
MAC_00973-qR	GTTATACTGAATACAAGAAGGAA	
MAC_08024-qF	GCCTCAAGTATATCCATTCA	147 bp
MAC_08024-qR	TGTATCCAGCATTCTCTTC	
MAC_08053-qF	TGGCACCATTACATATCG	110 bp
MAC_08053-qR	CAGAGTTGTTCTTCTTCATC	

Primer name	Sequence (5'-3')	Note
MAC_04751-qF	CAAGTTTTACACCGACAG	82 bp
MAC_04751-qR	CTCCTGCTTCTTCAGATC	
MAC_08084-qF	GAAGTAGACATCTGGAGTG	102 bp
MAC_08084-qR	GGTGATGATGGAGAACTG	
MAC_02310-qF	TGAGGAAGATGAGAATGAC	118 bp
MAC_02310-qR	GACAGTTGGAGGTGTATC	
MAC_02260-qF	CGAAAGCAGAGCATAATAC	191 bp
MAC_02260-qR	GTGGAAGATGACTTGACA	
MAC_02103-qF	CTCTTCACATCCACCATT	130 bp
MAC_02103-qR	GGCAATTATCCTCACTCA	
MAC_04776-qF	CGAGTCCAAGGAAGAATT	147 bp
MAC_04776-qR	ATGTTCGCATAGATGAGAG	
MAC_07045-qF	CCGATTCCTCATCTTCAA	137 bp
MAC_07045-qR	CGTCAATACACTGTTCAAC	
MAC_04310-qF	CAGCGAATTGTCTTGAAC	197 bp
MAC_04310-qR	GTGGTGGAAGTAGTCTTG	
MAC_01493-qF	TCTGTATCCTGGTGAGAA	180 bp
MAC_01493-qR	ATGGTATGCTGGTAGTATTC	
MAC_05385-qF	GTTCAGGCAGGTATAGATG	172 bp
MAC_05385-qR	ACTCAATGGCTTCTTCAG	
MAC_02654-qF	CCATGCAGTGAAAGAAAG	112 bp
MAC_02654-qR	GAATGTGTTTGCCTTCTG	
MAC_01225-qF	CGCCATTGTCTCTATTCT	126 bp

Primer name	Sequence (5'-3')	Note
MAC_01225-qR	ACGACTCCAAGATTGAAG	
MAC_06584-qF	ATGGACTCACTCAAGGAA	114 bp
MAC_06584-qR	GAGAGGTGCGTCAGATTT	
MAC_08011-qF	GGAAGAAGTTCAGGAGATT	98 bp
MAC_08011-qR	GTGATGCTATGTCTCTCTT	
MAC_09507-qF	ATGCAGTTCCTCGCTGTTGC	112 bp
MAC_09507-qR	ACTGGGGATTGCTGTAAAGACC	
MAC_04217-qF	CATTCACAATCACCAGGAT	81 bp
MAC_04217-qR	TTCTGCTGACGAGAGTAG	
MAC_07741-qF	TCAACCGAGAGGAGATTC	83 bp
MAC_07741-qR	GGTGAAGTGGATTGTGTT	
MAC_09703-qF	GAAATCTTGACTGTAAATG	96 bp
MAC_09703-qR	CGTAATAGAGGTAATGAC	
MAC_05034-qF	CTGATTGAGAAGGAAGTC	108 bp
MAC_05034-qR	AACCGTAATCTTGTCATC	
MAC_03969-qF	ATGGATCTCTGACGGATA	98 bp
MAC_03969-qR	GACTTGGACTTCTGTGAT	
MAC_04003-qF	TGCTGAGCCAGTATCTTG	80 bp
MAC_04003-qR	ACTGATAGAATGCCTCCG	
MAC_04467-qF	CTTATTGTCTACGACCATA	90 bp
MAC_04467-qR	CGAGTCATAGCAAGTAAT	
MAC_07994-qF	GATGATGTTACTCCAAGA	111 bp
MAC_07994-qR	ATTGTATCGGCTATTGAA	

Primer name	Sequence (5'-3')	Note
MAC_06320-qF	TTACTAACGAGCATCATAG	81 bp
MAC_06320-qR	GGCATATTCTGTGTCTAC	
MAC_09677-qF	CTGTTCTTTGGCATATTCC	80 bp
MAC_09677-qR	GTAGCGTAAGGTTGGTAG	
MAC_06613-qF	CAAGAACATTGCGGATAT	84 bp
MAC_06613-qR	CAGAACAGTGTCAAGAAT	
MAC_01182-qF	ACTACCTACAGAAGCAACA	133 bp
MAC_01182-qR	GCCGTCCACTACTCATT	
MAC_09277-qF	AACTCGTCAACCAGATAC	97 bp
MAC_09277-qR	ACTCTTGGACATTGTAGG	
MAC_04872-qF	GCACGGGATACATCTTCT	83 bp
MAC_04872-qR	GGCAATGAATGAGCAGAA	
MAC_07255-qF	AATTGGTTGGCTTGTTCA	110 bp
MAC_07255-qR	AAGAGTAAGTAAGGCGTAGA	
MAC_00224-qF	ATACTACGGCTCTGAATG	102 bp
MAC_00224-qR	CAGCATAAGCAATCATAAGA	
MAC_00986-qF	AGCGTGTATTTCTCCTTG	83 bp
MAC_00986-qR	CTTGCCTTGTTCGACTAA	
MAC_06364-qF	TTTCTTCCCACGAGGTTT	84 bp
MAC_06364-qR	GTGTAGGTGTCAAAGTCATC	
MAC_04557-qF	TTCCTCTTCTCCAGTGTT	112 bp
MAC_04557-qR	GAATAGTCCAGCAAGTGAT	
MAC_05902-qF	CATCAAGGAGACTCTTAC	120 bp

Primer name	Sequence (5'-3')	Note
MAC_05902-qR	TCCATATCAACAGACATT	
MAC_04556-qF	GCCTATGTGGTGTATGAA	89 bp
MAC_04556-qR	ATGACGCAGACAATGAAT	
MAC_08199-qF	CCCATCATAATCCGCTTT	95 bp
MAC_08199-qR	TGCCTAAGACTAACATTGAG	
MAC_08197-qF	GTGCTGTGATTGTTAGTG	81 bp
MAC_08197-qR	AGAGAATGGCAAGAATGA	

Table S2 Verification of DEGs results by RT-qPCR analysis

**Gene Symbol**	**Product name**	**DGE ΔMavib-1**	**RT-qPCR ΔMavib-1**	**DGE *Mavib-1* OE**	**RT-qPCR *Mavib-1* OE**
MAC_04217	heat shock protein 30	-3.49	-3.17	-1.93	-1.61
MAC_07741	putative cytochrome P450 3A7	-2.40	-2.13	-1.63	-1.37
MAC_09703	FAD binding domain-containing protein	-1.94	-1.26	3.07	3.57
MAC_05034	heat shock protein 101	-1.76	-1.42	-2.13	-4.72
MAC_03969	G-protein coupled receptor	-1.74	-1.54	-1.06	-0.53
MAC_04003	xylitol dehydrogenase	-1.68	-1.18	1.55	1.41
MAC_04467	laccase Lcc5	-1.58	-1.62	2.69	2.12
MAC_07994	alpha-1,2 mannosyltransferase KTR1	-1.57	-1.83	-1.66	-1.27
MAC_06320	aspartic proteinase	-1.39	-1.54	1.34	0.66
MAC_09677	ferrichrome-type siderophore transporter	-1.39	-1.21	1.50	1.13
MAC_06613	xenobiotic compound monooxygenase, DszA family, putative	-1.25	-1.41	1.31	1.66
MAC_01182	MedA	-1.20	-1.96	2.07	2.90
MAC_09277	glutamyl-tRNA(Gln) amidotransferase	-1.19	-1.11	1.27	1.66
MAC_04872	urea active transporter	1.12	0.84	-1.25	-1.06
MAC_07255	polysaccharide synthase Cps1, putative	1.29	0.86	2.00	2.24
MAC_00224	GAL4 & Fungal specific	-1.66	-1.53	-1.28	-0.13

Gene Symbol	Product name	DGE ∆*Mavib-1*	RT-qPCR ∆*Mavib-1*	DGE *Mavib-1* OE	RT-qPCR *Mavib-1* OE
	transcription factor domain containing protein				
MAC_00986	phenylacetyl-CoA ligase	-1.37	-0.94	1.27	2.27
MAC_06364	heat shock protein 30	-3.44	-3.56	-2.15	-2.43
MAC_04557	putative nucleoside-diphosphate-sugar epimerase	0.00	0.69	10.05	10.68
MAC_05902	carboxyphosphonoenolpyruvate phosphonomutase, putative	0.00	-0.30	7.68	7.97
MAC_04556	siderophore iron transporter, putative	-1.69	-0.62	6.35	7.56
MAC_08199	aldehyde dehydrogenase	0.25	0.34	5.96	6.11
MAC_08197	MFS monocarboxylate transporter, putative	-0.17	-0.25	5.89	6.16

Table S3 DEGs of Δ*Mavib-1* compared to WT cultured on SYA medium for 20 h

**Gene Symbol**	**Description**	**log_2(Δ*Mavib-1* / WT)**
**CAZyme**		
MAC_05562	alpha-galactosidase	-1.14
MAC_07255	polysaccharide synthase Cps1, putative	1.29
MAC_03417	acetyl-hydrolase	-1.35
MAC_06283	acid trehalase	-1.19
MAC_07994	alpha-1,2 mannosyltransferase KTR1	-1.57
MAC_09197	beta-1,6-glucanase	-1.34
MAC_02929	homoserine acetyltransferase family protein	-1.01
MAC_09703	isoamyl alcohol oxidase, putative	-1.94
MAC_07244	putative multicopperoxidase	1.08
**Carbon metabolism**		
MAC_09529	oxidoreductase, zinc-binding protein	-1.28
MAC_08191	ribose-phosphate pyrophosphokinase	1.14
MAC_02565	phosphoethanolamine transferase PIGF	-1.07
MAC_01637	acetoacetyl-CoA synthase	-1.12
MAC_04200	alcohol dehydrogenase, putative	-1.15
MAC_01403	hexose transport-related protein	-2.16
**Nitrogen metabolism**		
MAC_06383	aspartate carbamoyltransferase catalytic subunit	1.11
MAC_06029	ornithine decarboxylase	1.03
MAC_05125	S-adenosylmethionine decarboxylase proenzyme	1.09
MAC_04872	urea active transporter	1.12

Gene Symbol	Description	log_2(Δ*Mavib-1* / WT)
MAC_05344	urea transporter	1.01
MAC_07491	urease	1.00
MAC_03493	nitrite reductase	1.36
MAC_06321	NmrA family protein	-1.90
MAC_09768	Amino-acid permease inda1	1.03
MAC_05400	aspartate aminotransferase, putative	9.28
MAC_06320	aspartic proteinase	-1.39
MAC_09277	glutamyl-tRNA(Gln) amidotransferase	-1.19
**Lipid metabolism**		
MAC_02669	fatty acid desaturase	-1.28
MAC_09815	fatty acid-binding protein FABP, putative	-2.05
MAC_07120	cytochrome P450 52A11	-1.94
MAC_09276	cytochrome P450 52A12	-1.05
MAC_01100	cytochrome P450 oxidoreductase, putative	-1.10
**Signal transduction and gene transcription**		
MAC_04218	DNA repair protein RAD51	-1.12
MAC_09046	trypsin-related protease	-1.82
MAC_06833	trypsin-related protease	-1.10
MAC_00386	DNA topoisomerase III	1.02
MAC_02831	DNA-dependent RNA polymerase I subunit A43	1.06
MAC_07811	DNA-directed RNA polymerase I 135 kDa polypeptide	1.02
MAC_05663	RNA polymerase III subunit RPC82	1.04
MAC_05264	RNA-3'-phosphate cyclase family protein	1.12

Gene Symbol	Description	$\log_2$(Δ*Mavib-1* / WT)
MAC_01287	rRNA processing protein Bystin, putative	1.06
MAC_01993	Superoxide dismutase	1.09
MAC_03969	G-protein coupled receptor	-1.74
MAC_01182	MedA	-1.20
MAC_04517	bZIP transcription factor	1.80
MAC_00302	C2 domain protein	-1.00
MAC_08825	C2H2 finger domain protein FlbC	-1.35
MAC_03046	C2H2 finger domain protein, putative	-1.26
MAC_08573	C2H2 finger domain protein, putative	1.11
MAC_09257	C6 and C2H2 transcription factor RegA-like protein	-1.07
MAC_02667	C6 transcription factor, putative	-1.11
MAC_05515	C6 transcription factor, putative	1.01
MAC_04528	C6 zinc finger domain protein	-1.24
MAC_07846	calcium-transporting ATPase 3	-1.64
MAC_00224	GAL4 & Fungal specific transcription factor domain containing protein	-1.66
MAC_04865	swim zinc finger domain protein	-1.22
MAC_03911	AN1-type zinc finger protein	-1.04
MAC_08402	Cutinase transcription factor 1 beta	-1.05
MAC_04569	gag protein	-1.02
MAC_06589	putative DNA-binding protein	-1.48
MAC_04099	MYB DNA-binding domain-containing protein	-1.03
**Pathogenicity related**		
MAC_00987	adhesin protein Mad1	1.95

Gene Symbol	Description	log_2(Δ*Mavib-1* / WT)
MAC_07558	major allergen Asp f 2-like protein	2.15
MAC_01990	short chain dehydrogenase	-1.44
MAC_04315	putative ATP-dependent helicase	-1.24
MAC_00595	cytochrome P450, putative	-1.36
MAC_03401	YT521-B-like family protein	-1.16
MAC_00775	putative chloroperoxidase	-1.05
MAC_01566	MFS monosaccharide transporter, putative	-1.33
MAC_00237	MHYT domain signaling protein, putative	1.09
MAC_07386	ab-hydrolase associated lipase, putative	-1.09
MAC_09538	putative Ca2+/H+-exchanging protein	-1.46
MAC_05031	interferon-induced GTP-binding protein Mx	-1.69
MAC_05732	cytochrome P450	1.63
MAC_08514	flotillin domain containing protein	-1.76
MAC_00947	MFS transporter	1.14
MAC_04467	laccase Lcc5	-1.58
MAC_09362	phosphate-repressible phosphate permease	1.33
MAC_05040	candidapepsin-4 precursor	-2.49
MAC_07597	beta-lactamase, putative	-1.58
MAC_08908	glutaminyl-tRNA synthetase	1.62
MAC_03963	multidrug resistant protein	1.01
MAC_09677	ferrichrome-type siderophore transporter	-1.39
MAC_04887	putative aspartic endopeptidase	-1.16
MAC_07124	PAP2 domain containing protein	-1.48

Gene Symbol	Description	log2(Δ*Mavib-1* / WT)
MAC_01765	key lime pathogenicity protein	-1.48
MAC_04122	MFS transporter, putative	-1.10
MAC_00287	penicillin-binding protein, putative	-3.44
MAC_07309	putative P-type ATPase	-1.59
MAC_06859	MFS multidrug transporter, putative	-1.57
MAC_07357	cytochrome P450 3A17	-1.00
**Cell wall integrity**		
MAC_03694	WSC domain protein, putative	1.09
MAC_07327	chitinase	1.12
MAC_06488	glycine-rich cell wall structural protein 1	-1.01
**Stress response and detoxification**		
MAC_05034	heat shock protein 101	-1.76
MAC_04217	heat shock protein 30	-3.49
MAC_06364	heat shock protein 30	-3.44
MAC_07086	heat shock protein 78 precursor	-1.60
MAC_02364	Hsp40 co-chaperone Jid1, putative	-1.76
**Membrane protein**		
MAC_06214	integral membrane protein	-1.99
MAC_06538	integral membrane protein	-1.90
MAC_08540	integral membrane protein	-1.29
MAC_02040	ankyrin repeat containing protein	-1.27
MAC_02332	ankyrin repeat protein	-1.24
**Cellular transport**		

Gene Symbol	Description	$\log_2$(Δ*Mavib-1* / WT)
MAC_01878	ABC transporter (Adp1)	-1.23
MAC_00267	ABC-type Fe3+ transport system	-1.08
MAC_03697	ABC multidrug transporter Mdr1	-1.19
MAC_08884	allantoate transport protein	1.16
MAC_00825	anion exchange protein, putative	-1.09
**Other cellular metabolism**		
MAC_03200	3-beta hydroxysteroid dehydrogenase/isomerase family protein, putative	-1.15
MAC_02212	3-oxoacyl-(acyl carrier protein) reductase	-1.59
MAC_02812	60S ribosome biogenesis protein Mak11, putative	1.20
MAC_02449	amidase	1.07
MAC_00212	AMP-binding enzyme, putative	-1.12
MAC_04991	anthranilate synthase component II	-1.49
MAC_01012	ATP synthase gamma chain precursor	-1.08
MAC_00676	ATP-dependent RNA helicase DBP7, putative	1.00
MAC_01598	ATP-dependent RNA helicase dbp9	1.10
MAC_05846	benzoate 4-monooxygenase cytochrome P450	-1.05
MAC_05050	Brix domain containing protein	1.29
MAC_07655	conserved glycine-rich protein	-2.26
MAC_01196	cysteine synthase B, putative	1.23
MAC_00603	cysteine synthase K/M:Cysteine synthase B	1.65
MAC_06738	D-aminoacylase	-1.12
MAC_09268	delta 8-(E)-sphingolipid desaturase, putative	-2.71
MAC_04228	DRAP deaminase	-1.83

Gene Symbol	Description	log_2(Δ*Mavib-1* / WT)
MAC_01700	dual specificity phosphatase, putative	1.23
MAC_08416	DUF221 domain protein, putative	-1.25
MAC_01972	DUF500 domain protein	-1.51
MAC_09081	eukaryotic ribosome biogenesis protein 1	1.00
MAC_09358	extracellular dioxygenase, putative	-3.34
MAC_03642	extracellular serine-rich protein	-1.07
MAC_02143	extracellular serine-rich protein, putative	1.23
MAC_01118	FAD linked oxidase domain protein	-1.34
MAC_05270	ferric reductase transmembrane component, putative	-1.56
MAC_04988	feruloyl esterase B precursor, putative	-1.10
MAC_06853	flavohemoglobin	2.03
MAC_02061	GABA permease, putative	1.02
MAC_04028	gibberellin 20-oxidase, putative	-2.28
MAC_00081	glycerophosphodiester phosphodiesterase GDE1	1.13
MAC_02360	Glyoxalase/bleomycin resistance protein/dioxygenase	-2.00
MAC_09200	GNAT family acetyltransferase, putative	-1.02
MAC_04576	HMG box protein, putative	-1.21
MAC_07139	La domain family	1.03
MAC_02908	Leucine Rich Repeat domain protein	-1.03
MAC_01117	MFS monocarboxylate transporter, putative	-1.33
MAC_00551	Mitochondrial protein cyt-4	1.04
MAC_06616	oligopeptide transporter	1.14
MAC_06818	oligopeptide transporter OPT-like protein	-1.01

Gene Symbol	Description	log2(Δ*Mavib-1* / WT)
MAC_02365	PAF acetylhydrolase family protein	-2.27
MAC_02362	pantetheine-phosphate adenylyltransferase family protein	-1.06
MAC_00986	phenylacetyl-CoA ligase	-1.37
MAC_05950	plasma membrane calcium-transporting ATPase 2	-1.48
MAC_07325	PQQ repeat containing protein	1.24
MAC_00853	Pre-rRNA-processing protein ipi1	1.15
MAC_06592	proline oxidase PrnD	-1.15
MAC_07223	Pumilio-family RNA binding repeat protein	-1.20
MAC_03984	putative CHIP protein (carboxyl terminus of Hsc70-interacting protein)	-1.03
MAC_07741	putative cytochrome P450 3A7	-2.40
MAC_08753	putative fzr protein	-1.04
MAC_07245	putative iron transferase	1.54
MAC_03601	putative LTE1 protein	-1.27
MAC_07376	putative nucleoside-diphosphate-sugar epimerase	-1.23
MAC_01101	putative polyketide synthase protein	-1.17
MAC_01902	putative protein kinase	-1.12
MAC_09438	putative rna-binding protein fus/tls	-1.26
MAC_07473	pyridoxine	1.20
MAC_00381	QDE-2-interacting protein	-1.08
MAC_01768	riboflavine-aldehyde-forming enzyme	-1.37
MAC_01432	RTA1 domain protein, putative	-1.02
MAC_08291	serine peptidase, putative	-1.23

Gene Symbol	Description	log_2(Δ*Mavib-1* / WT)
MAC_01900	serine/threonine protein kinase, putative	-1.19
MAC_06605	short-chain dehydrogenases/reductase, putative	-1.48
MAC_00723	SNF2 family helicase/ATPase, putative	-1.08
MAC_01251	snoRNP assembly factor Naf1, putative	1.16
MAC_04418	srpk, putative	-2.26
MAC_03596	stomatin-like protein	-1.65
MAC_06739	sulfatase domain protein	-1.66
MAC_08642	sulfonate biosynthesis enzyme, putative	-1.29
MAC_08251	SWIRM domain-containing protein	-1.65
MAC_03313	transposase-like protein	-1.63
MAC_05232	TruB family pseudouridylate synthase containing protein	1.23
MAC_04822	U-box domain-containing protein	-1.46
MAC_07254	von Willebrand factor	-1.21
MAC_06613	xenobiotic compound monooxygenase, DszA family, putative	-1.25
MAC_04216	YT521-B-like splicing factor, putative	-1.72
MAC_05073	zinc-containing alcohol dehydrogenase superfamily protein	1.49
MAC_02279	dipeptidyl peptidase III	-1.41
**Unknown**		
MAC_08795	hypothetical protein	-2.30
MAC_04300	hypothetical protein	-2.26
MAC_07873	hypothetical protein	-2.20

Gene Symbol	Description	log$_2$(Δ*Mavib-1* / WT)
MAC_07534	hypothetical protein	-2.16
MAC_00652	hypothetical protein	-2.14
MAC_05220	hypothetical protein	-2.13
MAC_07886	hypothetical protein	-2.02
MAC_03029	hypothetical protein	-2.01
MAC_05271	hypothetical protein	-2.00
MAC_07160	hypothetical protein	-1.99
MAC_02185	hypothetical protein	-1.95
MAC_08454	hypothetical protein	-1.93
MAC_03214	hypothetical protein	-1.93
MAC_00278	hypothetical protein	-1.85
MAC_06322	hypothetical protein	-1.85
MAC_05450	hypothetical protein	-1.76
MAC_07632	hypothetical protein	-1.75
MAC_03022	hypothetical protein	-1.75
MAC_09762	hypothetical protein	-1.74
MAC_05428	hypothetical protein	-1.71
MAC_08975	hypothetical protein	-1.70
MAC_04003	hypothetical protein	-1.68
MAC_06767	hypothetical protein	-1.66
MAC_02363	hypothetical protein	-1.64
MAC_00389	hypothetical protein	-1.64
MAC_00556	hypothetical protein	-1.64

Gene Symbol	Description	$\log_2(\Delta$*Mavib-1* / WT)
MAC_03407	hypothetical protein	-1.62
MAC_07094	hypothetical protein	-1.61
MAC_04679	hypothetical protein	-1.60
MAC_02266	hypothetical protein	-1.60
MAC_02330	hypothetical protein	-1.55
MAC_08509	hypothetical protein	-1.51
MAC_08464	hypothetical protein	-1.47
MAC_03372	hypothetical protein	-1.43
MAC_06367	hypothetical protein	-1.40
MAC_00209	hypothetical protein	-1.39
MAC_08530	hypothetical protein	-1.36
MAC_06553	hypothetical protein	-1.35
MAC_07293	hypothetical protein	-1.34
MAC_05151	hypothetical protein	-1.33
MAC_03039	hypothetical protein	-1.32
MAC_01994	hypothetical protein	-1.30
MAC_04127	hypothetical protein	-1.29
MAC_09106	hypothetical protein	-1.29
MAC_01354	hypothetical protein	-1.28
MAC_05573	hypothetical protein	-1.27
MAC_07417	hypothetical protein	-1.26
MAC_05445	hypothetical protein	-1.25
MAC_01086	hypothetical protein	-1.25

**Gene Symbol**	**Description**	**log$_2$(Δ*Mavib-1* / WT)**
MAC_02361	hypothetical protein	-1.23
MAC_04727	hypothetical protein	-1.23
MAC_03096	hypothetical protein	-1.22
MAC_01769	hypothetical protein	-1.21
MAC_07670	hypothetical protein	-1.20
MAC_08505	hypothetical protein	-1.19
MAC_00294	hypothetical protein	-1.17
MAC_05020	hypothetical protein	-1.17
MAC_04322	hypothetical protein	-1.16
MAC_09283	hypothetical protein	-1.15
MAC_08164	hypothetical protein	-1.14
MAC_07288	hypothetical protein	-1.13
MAC_02638	hypothetical protein	-1.13
MAC_06604	hypothetical protein	-1.11
MAC_06148	hypothetical protein	-1.10
MAC_04227	hypothetical protein	-1.09
MAC_09581	hypothetical protein	-1.09
MAC_01851	hypothetical protein	-1.09
MAC_03241	hypothetical protein	-1.08
MAC_03959	hypothetical protein	-1.07
MAC_07101	hypothetical protein	-1.06
MAC_08396	hypothetical protein	-1.06
MAC_05993	hypothetical protein	-1.06

Gene Symbol	Description	$\log_2$(Δ*Mavib-1* / WT)
MAC_04561	hypothetical protein	-1.04
MAC_02096	hypothetical protein	-1.03
MAC_05236	hypothetical protein	-1.01
MAC_02662	hypothetical protein	-1.01
MAC_04780	hypothetical protein	-1.01
MAC_01912	hypothetical protein	-1.00
MAC_09177	hypothetical protein	-1.00
MAC_06504	hypothetical protein	1.02
MAC_06170	hypothetical protein	1.03
MAC_06328	hypothetical protein	1.03
MAC_07383	hypothetical protein	1.04
MAC_02832	hypothetical protein	1.04
MAC_02678	hypothetical protein	1.04
MAC_05729	hypothetical protein	1.05
MAC_00611	hypothetical protein	1.10
MAC_04761	hypothetical protein	1.11
MAC_06123	hypothetical protein	1.12
MAC_08357	hypothetical protein	1.13
MAC_03993	hypothetical protein	1.14
MAC_05109	hypothetical protein	1.14
MAC_01549	hypothetical protein	1.14
MAC_03111	hypothetical protein	1.19
MAC_08284	hypothetical protein	1.46

Gene Symbol	Description	log_2(Δ*Mavib-1* / WT)
MAC_05976	hypothetical protein	1.52
MAC_07661	hypothetical protein	1.99
MAC_07182	hypothetical protein	2.16
MAC_02556	hypothetical protein	2.39
MAC_05319	hypothetical protein	2.79
MAC_00391	hypothetical protein	3.49
MAC_07499	hypothetical protein	6.61
MAC_07302	hypothetical protein	-1.63

Table S4 DEGs of *Mavib-1* in OE compared to WT cultured on SYA medium for 20 h

Gene Symbol	Description	log_2(OE/WT)
**CAZyme**		
MAC_01220	alcohol dehydrogenase	1.65
MAC_09392	1,2-a-D-mannosidase	1.12
MAC_03417	acetyl-hydrolase	-1.05
MAC_07113	alpha/beta hydrolase fold domain containing protein	1.62
MAC_07994	alpha-1,2 mannosyltransferase KTR1	-1.66
MAC_00623	beta-glucosidase	2.71
MAC_08465	Cel3b putative secreted beta-glucosidase	-1.13
MAC_06612	Cel5b putative endoglucanase	1.75
MAC_08916	cell surface protein	2.14
MAC_00160	cell wall glycosyl hydrolase YteR, putative	2.16
MAC_04381	chitinase	-1.01
MAC_08492	chitinase	-1.09
MAC_07327	chitinase	-1.25
MAC_04884	chitinase 18-18	1.12
MAC_00345	chitinase 18-3	-1.88
MAC_04294	chitosanase	2.77
MAC_05829	choline dehydrogenase, putative	1.80
MAC_07331	cholinesterase	-1.84
MAC_02142	endo alpha-1,4 polygalactosaminidase precusor	1.40
MAC_02571	endoglucanase, putative	-1.34
MAC_00550	exo-beta-1,3-glucanase	1.52

Gene Symbol	Description	log_2(OE/WT)
MAC_01988	exo-polygalacturonase, putative	-1.06
MAC_06333	FAD binding domain protein	1.38
MAC_09703	FAD binding domain-containing protein	3.07
MAC_02934	glucose-methanol-choline oxidoreductase	2.22
MAC_01372	Glycosyl hydrolase family 16 protein	-1.06
MAC_02139	glycosyl transferase, putative	2.59
MAC_06157	glycosyl transferase, putative	-1.13
MAC_02882	glycosyltransferase	3.92
MAC_06700	GMC oxidoreductase	3.23
MAC_05784	lipase/esterase family protein, putative	3.92
MAC_02090	meiotically up-regulated protein	1.74
MAC_02485	N,O-diacetyl muramidase, putative	5.17
MAC_06282	N-acetylglucosaminidase	1.43
MAC_01567	NAD binding Rossmann fold oxidoreductase, putative	-1.35
MAC_02088	Phospholipase/Carboxylesterase family protein	1.66
MAC_07255	polysaccharide synthase Cps1, putative	2.00
MAC_04394	putative beta 1,3-galactosyltransferase polypeptide 1	1.06
MAC_06016	putative beta-glucosidase 1 precursor	2.58
MAC_08411	putative endochitinase CHI2	-1.86
MAC_06143	putative glyoxal oxidase precursor	2.08
**Carbon metabolism**		
MAC_01403	hexose transport-related protein	-2.16
MAC_01989	glucose transporter	1.40

Gene Symbol	Description	$\log_2$(OE/WT)
MAC_01225	glucose transporter-like protein	-1.11
MAC_02951	sugar transporter	1.97
MAC_04267	sugar transporter family protein	1.07
MAC_03497	glucosamine-6-phosphate deaminase	-1.20
MAC_04299	UDP-glucose 6-dehydrogenase	-1.27
**Nitrogen metabolism**		
MAC_04872	urea active transporter	-1.25
MAC_09277	glutamyl-tRNA(Gln) amidotransferase	1.27
MAC_08898	glutamate decarboxylase, putative	3.65
MAC_09524	glutathione S-transferase	-1.23
MAC_05310	glutathione-dependent formaldehyde-activating	1.11
MAC_06320	aspartic proteinase	1.34
MAC_00229	amino acid permease, putative	3.14
MAC_09782	amino acid transporter arg-13	1.42
MAC_03365	amino acid transporter, putative	1.24
MAC_00316	Amino-acid permease inda1	4.02
MAC_04079	Amino-acid permease inda1	-1.55
MAC_08200	tryptophanyl-tRNA synthetase	5.86
**Lipid metabolism**		
MAC_02669	fatty acid desaturase	-1.24
MAC_06765	fatty acid synthase beta subunit dehydratase	-1.01
MAC_05732	cytochrome P450	1.36
MAC_00512	cytochrome P450 ClCP1	1.71

Gene Symbol	Description	log_2(OE/WT)
MAC_04407	Cytochrome P450 family protein	5.79
MAC_06623	cytochrome P450 phenylacetate 2-hydroxylase, putative	5.41
MAC_01110	cytochrome P450, putative	-1.29
**Signal transduction and gene transcription**		
MAC_08931	transcription factor atf21	1.61
MAC_06768	transcriptional activator xlnR	-1.95
MAC_03710	transcriptional regulator, putative	1.36
MAC_04517	bZIP transcription factor	1.07
MAC_02987	bZIP transcription factor, putative	1.06
MAC_00734	C2H2 transcription factor	-1.01
MAC_04110	C6 transcription factor, putative	3.63
MAC_08121	C6 transcription factor, putative	1.00
MAC_02136	C6 zinc finger domain protein	4.02
MAC_00655	C6 zinc finger domain-containing protein	-3.24
MAC_04218	DNA repair protein RAD51	-1.11
MAC_03048	Fungal Zn binuclear cluster domain containing protein	3.36
MAC_00224	GAL4 & Fungal specific transcription factor domain containing protein	-1.28
MAC_03969	G-protein coupled receptor	-1.06
MAC_01182	MedA	2.07
MAC_04665	putative ZIP zinc transporter	1.05
MAC_09046	trypsin-related protease	-1.69
MAC_06833	trypsin-related protease	-1.80
**Pathogenicity related**		

Gene Symbol	Description	$\log_2$(OE/WT)
MAC_07558	major allergen Asp f 2-like protein	1.41
MAC_01990	short chain dehydrogenase	-1.23
MAC_08496	methyl transferase-like protein	1.26
MAC_07470	subtilisin-like serine protease PR1C	4.07
MAC_08198	pyoverdine/dityrosine biosynthesis protein, putative	6.19
MAC_05902	carboxyphosphonoenolpyruvate phosphonomutase, putative	7.68
MAC_02342	steroid monooxygenase, putative	1.04
MAC_08215	O-methyltransferase, putative	3.44
MAC_00588	2-epi-5-epi-valiolone synthase	4.09
MAC_05087	sexual differentiation process protein isp4, putative	1.96
MAC_02895	uracil permease	-1.68
MAC_04256	efflux pump antibiotic resistance protein, putative	-1.01
MAC_08199	aldehyde dehydrogenase	5.96
MAC_04550	Major Facilitator Superfamily protein	2.77
MAC_08197	MFS monocarboxylate transporter, putative	5.89
MAC_02196	NmrA family transcriptional regulator	-1.63
MAC_08514	flotillin domain containing protein	1.08
MAC_02260	GTPase activating protein (Gyp3), putative	-1.00
MAC_06567	allantoate permease, putative	4.51
MAC_09273	putative allantoate permease of the major facilitator superfamily	1.80
MAC_05384	laccase	-2.20
MAC_04408	ABC transporter	3.09
MAC_02894	extracellular phospholipase C	3.82

Gene Symbol	Description	$\log_2$(OE/WT)
MAC_00210	flavin containing polyamine oxidase, putative	2.02
MAC_04467	laccase Lcc5	2.69
MAC_05171	phosphate permease	-1.28
MAC_02353	sodium/phosphate symporter, putative	1.41
MAC_00955	3-isopropylmalate dehydrogenase	-2.62
MAC_05040	candidapepsin-4 precursor	-1.27
MAC_05605	perilipin-like protein	1.75
MAC_07597	beta-lactamase, putative	-1.33
MAC_05385	conidial pigment polyketide synthase PksP/Alb1	-2.28
MAC_04410	lysine amidinotransferase	4.15
MAC_09160	MFS phospholipid transporter Git1	1.30
MAC_09677	ferrichrome-type siderophore transporter	1.50
MAC_08294	siderophore iron transporter	1.23
MAC_04556	siderophore iron transporter, putative	6.35
MAC_00287	penicillin-binding protein, putative	-2.18
MAC_06859	MFS multidrug transporter, putative	-1.95
MAC_02091	acyltransferase, putative	1.44
MAC_04118	alanine racemase	2.06
MAC_08656	putative glyoxal oxidase precursor	5.38
MAC_00987	adhesin protein Mad1	3.37
MAC_05411	subtilisin-like protease	-1.12
**Cell wall integrity**		
MAC_04903	cell wall galactomannoprotein Mp2/allergen F17-like protein	2.84

Gene Symbol	Description	log_2(OE/WT)
MAC_06850	cell wall protein	1.64
MAC_07330	Hydrophobin-like protein ssgA	2.63
**Stress response and detoxification**		
MAC_05034	heat shock protein 101	-2.13
MAC_04217	heat shock protein 30	-1.93
MAC_06364	heat shock protein 30	-2.15
MAC_07086	heat shock protein 78 precursor	-1.71
MAC_00155	Hsp70 family chaperone, putative	5.65
**Membrane protein**		
MAC_00494	integral membrane family protein	2.05
MAC_00161	integral membrane protein	5.63
MAC_07739	integral membrane protein	2.29
MAC_04572	integral membrane protein	2.23
MAC_03905	integral membrane protein	1.35
MAC_04723	integral membrane protein	1.30
MAC_03331	integral membrane protein	-1.01
MAC_06568	integral membrane protein, putative	5.84
MAC_05731	integral membrane protein, putative	2.98
MAC_09015	integral membrane protein, putative	1.72
MAC_09359	integral membrane protein, putative	-1.35
MAC_08930	aquaporin	-1.56
MAC_02576	transmembrane glycoprotein, putative	-1.03
MAC_05991	Plasma membrane proteolipid 3	1.01

Gene Symbol	Description	log_2(OE/WT)
MAC_08765	pheromone-regulated multispanning membrane protein Prm1, putative	-1.10
**Cellular transport**		
MAC_04282	transporter-like protein	2.37
MAC_05849	cation transport ATPase	-1.76
MAC_09357	chloride channel protein, putative	-1.15
MAC_06066	siderophore iron transporter mirB	1.05
MAC_07963	vacuolar calcium ion transporter, putative	1.10
MAC_08868	MFS transporter	2.87
MAC_06625	MFS transporter (Mch2), putative	4.39
MAC_04724	MFS transporter, putative	1.79
MAC_06963	MFS transporter, putative	1.12
MAC_09623	ABC transporter, putative	2.25
MAC_07962	calcium ion transporter Vcx1, putative	2.08
MAC_00876	oligopeptide transporter OPT-like protein	1.07
MAC_08276	outer membrane autotransporter	1.70
MAC_05825	vitamin B6 transporter, putative	2.92
MAC_01249	sulfate permease II	1.84
**Other cellular metabolism**		
MAC_02365	PAF acetylhydrolase family protein	-1.41
MAC_00986	phenylacetyl-CoA ligase	1.27
MAC_06592	proline oxidase PrnD	-1.40
MAC_07223	Pumilio-family RNA binding repeat protein	-1.08
MAC_07741	putative cytochrome P450 3A7	-1.63

Gene Symbol	Description	$\log_2$(OE/WT)
MAC_01101	putative polyketide synthase protein	-1.02
MAC_01768	riboflavine-aldehyde-forming enzyme	-1.39
MAC_08291	serine peptidase, putative	-1.38
MAC_03313	transposase-like protein	-1.38
MAC_06613	xenobiotic compound monooxygenase, DszA family, putative	1.31
MAC_04576	HMG box protein, putative	-1.15
MAC_09200	GNAT family acetyltransferase, putative	-1.19
MAC_04028	gibberellin 20-oxidase, putative	-1.90
MAC_09358	extracellular dioxygenase, putative	-3.73
MAC_09232	extracellular lipase	-1.67
MAC_03642	extracellular serine-rich protein	-1.04
MAC_02143	extracellular serine-rich protein, putative	2.32
MAC_04228	DRAP deaminase	-1.68
MAC_01700	dual specificity phosphatase, putative	1.01
MAC_02279	dipeptidyl peptidase III	-1.10
MAC_01196	cysteine synthase B, putative	-1.08
MAC_02449	amidase	-1.47
MAC_01012	6-phosphogluconate dehydrogenase, NAD-binding protein	-1.24
MAC_07097	kinesin	1.19
MAC_02819	2,3-dihydroxybenzoic acid decarboxylase dhbD	1.09
MAC_06281	2-nitropropane dioxygenase	1.26
MAC_04117	2OG-Fe(II) oxygenase family oxidoreductase, putative	1.54
MAC_09383	2OG-Fe(II) oxygenase family oxidoreductase, putative	1.48

Gene Symbol	Description	log_2(OE/WT)
MAC_03200	3-beta hydroxysteroid dehydrogenase/isomerase family protein, putative	-1.17
MAC_08104	3-octaprenyl-4-hydroxybenzoate carboxy-lyase, putative	-1.92
MAC_01745	3'-tRNA processing endoribonuclease	-1.41
MAC_05007	4-aminobutyrate aminotransferase	-1.32
MAC_01226	ABC drug exporter AtrF	1.33
MAC_06829	acetamidase	-1.94
MAC_08695	acetolactate synthase	1.18
MAC_01859	acetylase, putative	-2.22
MAC_02483	acid phosphatase precursor	1.89
MAC_08531	amidohydrolase 2	3.21
MAC_03832	amidohydrolase family protein	1.17
MAC_06735	arrestin	1.05
MAC_03628	ATP-citrate synthase subunit 1	-1.24
MAC_02852	beta-lactamase	1.31
MAC_01323	branched-chain amino acid aminotransferase, putative	-1.30
MAC_05025	calcineurin-like phosphoesterase, putative	2.00
MAC_09459	CAMK family protein kinase	1.63
MAC_03712	catalase	1.18
MAC_04554	citrinin biosynthesis transcriptional activator CtnR	6.39
MAC_04340	collagen-like protein Mcl1	1.04
MAC_05828	Cupin family protein	2.76
MAC_05826	Cupin family protein	2.00
MAC_05903	cysteine desulfurase	3.86

Gene Symbol	Description	log_2(OE/WT)
MAC_07038	dicarboxylic amino acid permease	1.29
MAC_07011	dienelactone hydrolase family protein	2.80
MAC_01548	DOC family protein	2.02
MAC_05901	DUF1275 domain protein	3.87
MAC_06624	DUF636 domain protein	4.08
MAC_00837	DUF718 domain protein	1.24
MAC_00176	DUF895 domain membrane protein	1.03
MAC_07838	enoyl-CoA hydratase/isomerase family protein	1.03
MAC_08796	exo-1,3-beta-D-glucanase	1.73
MAC_05743	farnesyl pyrophosphate synthetase 1	2.41
MAC_08103	flavoprotein family	-1.35
MAC_08932	formate dehydrogenase	2.27
MAC_08871	general amino acid permease AGP3	-1.01
MAC_02718	GNAT family acetyltransferase	-1.15
MAC_03099	GNS1/SUR4 family protein	-1.01
MAC_04771	GPI anchored protein, putative	1.06
MAC_01777	Guanyl-specific ribonuclease F1	-1.35
MAC_01563	H /K ATPase alpha subunit, putative	2.56
MAC_09212	heme-binding peroxidase	1.30
MAC_02133	homogentisate 1,2-dioxygenase	-2.78
MAC_03949	HPP family protein	1.54
MAC_09686	hscarg dehydrogenase, putative	2.66
MAC_09348	hydroxyisocaproate dehydrogenase	-1.21

Gene Symbol	Description	$\log_2$(OE/WT)
MAC_07816	Inorganic pyrophosphatase	1.63
MAC_05586	isochorismatase family hydrolase, putative	-1.22
MAC_03907	isoflavone reductase family protein	2.53
MAC_09103	lactam utilization protein LamB	2.08
MAC_02947	lactonohydrolase	1.14
MAC_09146	late sexual development protein	-2.63
MAC_00249	LEA domain protein	1.10
MAC_04553	leucoanthocyanidin dioxygenase, putative	8.86
MAC_06024	lipase	1.11
MAC_04378	magnesium-translocating P-type ATPase family protein	1.70
MAC_08022	malate dehydrogenase	1.33
MAC_00187	Maleylacetate reductase, putative	5.23
MAC_01408	mannosylphosphate transferase	-2.16
MAC_04112	metallo-beta-lactamase domain protein	2.23
MAC_07747	methylase involved in ubiquinone/menaquinone biosynthesis	-1.82
MAC_01539	monooxygenase, putative	3.54
MAC_04141	NAD dependent epimerase/dehydratase family protein	1.51
MAC_00077	NAD dependent epimerase/dehydratase, putative	1.20
MAC_05552	NADH oxidase	1.16
MAC_03488	neutral ceramidase precursor	1.23
MAC_06296	NlpC/P60-like cell-wall peptidase	3.81
MAC_01451	NlpC/P60-like cell-wall peptidase	3.34
MAC_09811	O-methyltransferase, putative	2.25

Gene Symbol	Description	log_2(OE/WT)
MAC_06944	P450 monooxygenase	3.53
MAC_09557	pathway-specific nitrogen regulator	1.61
MAC_01190	penicillopepsin	-1.20
MAC_02150	peptidase family T4 protein	2.06
MAC_02278	pfs domain-containing protein	-1.40
MAC_00781	phosphatidyl synthase	-1.29
MAC_04466	phosphoglycerate mutase family protein, putative	4.06
MAC_05098	phosphotransferase enzyme family protein	1.03
MAC_09553	phytanoyl-CoA dioxygenase	3.69
MAC_03162	phytanoyl-CoA dioxygenase	1.18
MAC_09175	protein CCC1, putative	1.58
MAC_02249	protein kinase-like protein	1.36
MAC_07571	Putative cryptochrome DASH	1.07
MAC_06615	putative cyclic nucleotide gated channel beta 1	1.00
MAC_02896	putative DCG1 protein	-2.22
MAC_03964	putative D-hydroxyacid dehydrogenase	1.52
MAC_08692	putative glyoxalase family protein	1.20
MAC_03198	putative histidine acid phosphatase	2.37
MAC_04557	putative nucleoside-diphosphate-sugar epimerase	10.05
MAC_08317	putative sialidase	-1.04
MAC_00602	putative tetracycline efflux protein (otrb)	1.04
MAC_01858	pyridine nucleotide-disulfide oxidoreductase AMID-like, putative	1.15
MAC_05442	pyruvate dehydrogenase, putative	1.56

Gene Symbol	Description	log_2(OE/WT)
MAC_06618	retinol dehydrogenase 8	-1.15
MAC_03447	sarcosine oxidase	-1.34
MAC_04455	ser/Thr protein phosphatase superfamily	1.05
MAC_02763	serine-type carboxypeptidase	5.79
MAC_04682	serine-type carboxypeptidase	2.07
MAC_06414	SH3 domain containing protein	1.02
MAC_01414	short chain dehydrogenase	-1.19
MAC_07623	sulfatase	-1.52
MAC_06569	thiamine pyrophosphate enzyme	1.19
MAC_09676	ThiJ/PfpI family protein	1.04
MAC_02186	thiol methyltransferase, putative	4.33
MAC_01562	transposase	1.78
MAC_08412	TRI14-like protein	-1.07
MAC_04360	tripsin, putative	-1.22
MAC_05716	tyrosinase, putative	1.97
MAC_05709	uracil permease, putative	3.84
MAC_04549	YCII-related domain protein	2.61
MAC_08195	zinc-binding oxidoreductase ToxD, putative	4.05
**Unknown**		
MAC_02137	hypothetical protein	6.53
MAC_08201	hypothetical protein	6.02
MAC_08196	hypothetical protein	5.83
MAC_04973	hypothetical protein	5.31

Gene Symbol	Description	$log_2(OE/WT)$
MAC_05095	hypothetical protein	4.95
MAC_06626	hypothetical protein	4.83
MAC_02957	hypothetical protein	4.56
MAC_05441	hypothetical protein	4.28
MAC_05835	hypothetical protein	3.99
MAC_01123	hypothetical protein	3.50
MAC_02670	hypothetical protein	3.39
MAC_04111	hypothetical protein	3.35
MAC_08447	hypothetical protein	3.18
MAC_02602	hypothetical protein	3.17
MAC_04417	hypothetical protein	3.15
MAC_05908	hypothetical protein	3.07
MAC_00584	hypothetical protein	2.93
MAC_00611	hypothetical protein	2.93
MAC_07302	hypothetical protein	2.78
MAC_08505	hypothetical protein	2.74
MAC_07882	hypothetical protein	2.50
MAC_02330	hypothetical protein	2.40
MAC_01540	hypothetical protein	2.35
MAC_09732	hypothetical protein	2.30
MAC_07186	hypothetical protein	2.27
MAC_03362	hypothetical protein	2.19
MAC_04283	hypothetical protein	2.17

Gene Symbol	Description	log_2(OE/WT)
MAC_05630	hypothetical protein	2.16
MAC_07526	hypothetical protein	2.14
MAC_04005	hypothetical protein	2.06
MAC_01934	hypothetical protein	2.05
MAC_02089	hypothetical protein	2.02
MAC_05629	hypothetical protein	1.98
MAC_09274	hypothetical protein	1.97
MAC_08795	hypothetical protein	1.91
MAC_01150	hypothetical protein	1.90
MAC_02486	hypothetical protein	1.88
MAC_04496	hypothetical protein	1.88
MAC_05640	hypothetical protein	1.86
MAC_02105	hypothetical protein	1.84
MAC_06604	hypothetical protein	1.81
MAC_07559	hypothetical protein	1.80
MAC_04833	hypothetical protein	1.79
MAC_07182	hypothetical protein	1.76
MAC_02992	hypothetical protein	1.75
MAC_09694	hypothetical protein	1.75
MAC_08708	hypothetical protein	1.73
MAC_07839	hypothetical protein	1.72
MAC_06367	hypothetical protein	1.69
MAC_04420	hypothetical protein	1.69

Gene Symbol	Description	$\log_2$(OE/WT)
MAC_09057	hypothetical protein	1.61
MAC_04137	hypothetical protein	1.57
MAC_04003	hypothetical protein	1.55
MAC_07844	hypothetical protein	1.54
MAC_09642	hypothetical protein	1.54
MAC_09630	hypothetical protein	1.53
MAC_03252	hypothetical protein	1.52
MAC_03311	hypothetical protein	1.51
MAC_02059	hypothetical protein	1.49
MAC_01375	hypothetical protein	1.48
MAC_04761	hypothetical protein	1.48
MAC_04763	hypothetical protein	1.47
MAC_07671	hypothetical protein	1.45
MAC_06083	hypothetical protein	1.44
MAC_02242	hypothetical protein	1.43
MAC_00585	hypothetical protein	1.41
MAC_09283	hypothetical protein	1.41
MAC_06328	hypothetical protein	1.39
MAC_01713	hypothetical protein	1.38
MAC_04929	hypothetical protein	1.37
MAC_03312	hypothetical protein	1.36
MAC_00246	hypothetical protein	1.34
MAC_07842	hypothetical protein	1.33

Gene Symbol	Description	log_2(OE/WT)
MAC_07383	hypothetical protein	1.33
MAC_02073	hypothetical protein	1.32
MAC_05606	hypothetical protein	1.31
MAC_01086	hypothetical protein	1.31
MAC_03674	hypothetical protein	1.31
MAC_03004	hypothetical protein	1.30
MAC_06597	hypothetical protein	1.30
MAC_07074	hypothetical protein	1.30
MAC_04278	hypothetical protein	1.28
MAC_04190	hypothetical protein	1.28
MAC_09004	hypothetical protein	1.26
MAC_04638	hypothetical protein	1.25
MAC_03253	hypothetical protein	1.25
MAC_03190	hypothetical protein	1.24
MAC_03005	hypothetical protein	1.22
MAC_09354	hypothetical protein	1.19
MAC_05737	hypothetical protein	1.17
MAC_06932	hypothetical protein	1.17
MAC_05091	hypothetical protein	1.16
MAC_00937	hypothetical protein	1.14
MAC_08894	hypothetical protein	1.14
MAC_02306	hypothetical protein	1.12
MAC_06378	hypothetical protein	1.12

Gene Symbol	Description	log_2(OE/WT)
MAC_05976	hypothetical protein	1.12
MAC_00214	hypothetical protein	1.10
MAC_00066	hypothetical protein	1.10
MAC_06323	hypothetical protein	1.10
MAC_08541	hypothetical protein	1.10
MAC_08279	hypothetical protein	1.09
MAC_05725	hypothetical protein	1.08
MAC_07236	hypothetical protein	1.08
MAC_08031	hypothetical protein	1.08
MAC_03366	hypothetical protein	1.08
MAC_09713	hypothetical protein	1.07
MAC_05621	hypothetical protein	1.05
MAC_02751	hypothetical protein	1.05
MAC_07012	hypothetical protein	1.04
MAC_02345	hypothetical protein	1.04
MAC_03449	hypothetical protein	1.04
MAC_01738	hypothetical protein	1.04
MAC_00834	hypothetical protein	1.03
MAC_07758	hypothetical protein	1.02
MAC_06160	hypothetical protein	1.01
MAC_08375	hypothetical protein	1.01
MAC_07515	hypothetical protein	1.00
MAC_08845	hypothetical protein	1.00

Gene Symbol	Description	log_2(OE/WT)
MAC_09762	hypothetical protein	-1.01
MAC_04883	hypothetical protein	-1.02
MAC_02452	hypothetical protein	-1.03
MAC_07101	hypothetical protein	-1.04
MAC_05022	hypothetical protein	-1.04
MAC_06391	hypothetical protein	-1.05
MAC_06072	hypothetical protein	-1.05
MAC_09489	hypothetical protein	-1.07
MAC_03096	hypothetical protein	-1.07
MAC_05320	hypothetical protein	-1.08
MAC_02653	hypothetical protein	-1.08
MAC_03407	hypothetical protein	-1.08
MAC_03560	hypothetical protein	-1.08
MAC_02857	hypothetical protein	-1.08
MAC_05993	hypothetical protein	-1.10
MAC_07745	hypothetical protein	-1.11
MAC_01276	hypothetical protein	-1.12
MAC_04127	hypothetical protein	-1.13
MAC_06553	hypothetical protein	-1.14
MAC_04679	hypothetical protein	-1.14
MAC_00278	hypothetical protein	-1.15
MAC_06239	hypothetical protein	-1.16
MAC_05427	hypothetical protein	-1.18

Gene Symbol	Description	log_2(OE/WT)
MAC_04401	hypothetical protein	-1.18
MAC_07098	hypothetical protein	-1.21
MAC_01354	hypothetical protein	-1.23
MAC_01851	hypothetical protein	-1.23
MAC_00194	hypothetical protein	-1.23
MAC_08464	hypothetical protein	-1.23
MAC_02096	hypothetical protein	-1.24
MAC_03214	hypothetical protein	-1.25
MAC_00556	hypothetical protein	-1.25
MAC_04620	hypothetical protein	-1.25
MAC_01714	hypothetical protein	-1.28
MAC_09312	hypothetical protein	-1.28
MAC_07670	hypothetical protein	-1.29
MAC_03039	hypothetical protein	-1.30
MAC_02266	hypothetical protein	-1.32
MAC_03676	hypothetical protein	-1.32
MAC_03372	hypothetical protein	-1.35
MAC_07619	hypothetical protein	-1.36
MAC_02043	hypothetical protein	-1.38
MAC_06674	hypothetical protein	-1.40
MAC_08509	hypothetical protein	-1.45
MAC_08954	hypothetical protein	-1.46
MAC_05748	hypothetical protein	-1.47

Gene Symbol	Description	log_2(OE/WT)
MAC_03029	hypothetical protein	-1.49
MAC_08315	hypothetical protein	-1.52
MAC_07632	hypothetical protein	-1.60
MAC_07744	hypothetical protein	-1.63
MAC_07160	hypothetical protein	-1.68
MAC_05927	hypothetical protein	-1.70
MAC_07534	hypothetical protein	-1.70
MAC_05220	hypothetical protein	-1.71
MAC_06538	hypothetical protein	-1.83
MAC_00672	hypothetical protein	-2.03
MAC_05271	hypothetical protein	-2.05
MAC_06767	hypothetical protein	-2.46
MAC_04300	hypothetical protein	-2.46
MAC_07886	hypothetical protein	-2.51
MAC_07094	hypothetical protein	-2.54
MAC_09485	hypothetical protein	-2.58

Table S5 DEGs with opposite changing trends of transcription in Δ*Mavib-1* and OE

Gene ID	Products name	$\log_2$(Δ*Mavib-1*/ WT)	$\log_2$(OE / WT)
MAC_09703	isoamyl alcohol oxidase, putative	-1.94	3.07
MAC_07302	hypothetical protein	-1.63	2.78
MAC_08505	hypothetical protein	-1.19	2.74
MAC_04467	laccase	-1.58	2.69
MAC_02330	hypothetical protein	-1.55	2.40
MAC_01182	MedA	-1.20	2.07
MAC_08795	hypothetical protein	-2.30	1.91
MAC_06604	hypothetical protein	-1.11	1.81
MAC_06367	hypothetical protein	-1.40	1.69
MAC_04003	xylitol dehydrogenase	-1.68	1.55
MAC_09677	siderochrome-iron transporter Sit1	-1.39	1.50
MAC_09283	hypothetical protein	-1.15	1.41
MAC_06320	aspartic proteinase	-1.39	1.34
MAC_01086	hypothetical protein	-1.25	1.31
MAC_06613	monooxygenase, DszA family, putative	-1.25	1.31
MAC_09277	glutamyl-tRNA (Gln) amidotransferase	-1.19	1.27
MAC_00986	phenylacetyl-CoA ligase	-1.37	1.27
MAC_08514	flotillin domain containing protein	-1.76	1.08
MAC_02449	Amidase	1.07	-1.47
MAC_04872	urea active transporter	1.12	-1.25

MAC_07327	chitinase	1.12	-1.25
MAC_01196	cysteine synthase B, putative	1.23	-1.08

Table S6 CAZyme in DEGs of Δ*Mavib-1*

**Gene ID**	**Products name**	**log$_2$(Δ*Mavib-1*/WT)**	**log$_2$(OE/WT)**	**Enzyme**	**Family**
MAC_09703	FAD binding domain-containing protein	-1.94	3.07	AA	AA7
MAC_07994	alpha-1,2 mannosyltransferase KTR1	-1.57	-1.66	GT	GT15
MAC_03417	acetyl-hydrolase	-1.35	-1.05	CE	CE10
MAC_09197	beta-1,6-glucanase	-1.34	0.98	GH	GH5
MAC_06283	acid trehalase	-1.19	-0.51	GH	GH65
MAC_05562	alpha-galactosidase	-1.14	-0.66	GH	GH36
MAC_02929	homoserine acetyltransferase family protein	-1.01	-0.57	CE	CE1
MAC_07244	putative multicopperoxidase	1.08	-0.03	AA	AA1
MAC_07255	polysaccharide synthase Cps1, putative	1.29	2.00	GT	GT21

Table S7 CAZyme in DEGs of OE

Gene Symbol	Products name	log_2(Δ*Mavib-1* / WT)	log_2(OE / WT)	Enzyme	Family
MAC_01372	N,O-diacetyl muramidase, putative	-0.49	5.17	GH	GH25
MAC_01567	lipase/esterase family protein, putative	-0.50	3.92	CE	CE10
MAC_02571	glycosyltransferase	-0.70	3.92	GT	GT1
MAC_04884	GMC oxidoreductase	-1.09	3.23	AA	AA3
MAC_00550	FAD binding domain-containing protein	-1.94	3.07	AA	AA7
MAC_03417	chitosanase	0.27	2.77	GH	GH75
MAC_01988	beta-glucosidase	0.51	2.71	GH	GH1
MAC_08492	glycosyl transferase, putative	0.15	2.59	GT	GT4
MAC_08411	putative beta-glucosidase 1 precursor	0.05	2.58	GH	GH3
MAC_04381	glucose-methanol-choline oxidoreductase	-0.33	2.22	AA	AA3
MAC_07331	cell wall glycosyl hydrolase YteR, putative	-0.67	2.16	GH	GH105
MAC_02882	cell surface protein	0.42	2.14	GH	GH135
MAC_05829	putative glyoxal oxidase precursor	0.21	2.08	AA	AA5
MAC_08916	polysaccharide synthase Cps1, putative	1.29	2.00	GT	GT21
MAC_06016	choline dehydrogenase, putative	0.07	1.80	AA	AA3
MAC_06612	Cel5b putative endoglucanase	-0.74	1.75	GH	GH5
MAC_07255	meiotically up-regulated protein	0.79	1.74	GT	GT8

Gene Symbol	Products name	log2(Δ*Mavib-1* / WT)	log2(OE / WT)	Enzyme	Family
MAC_02934	Phospholipase/Carboxylesterase family protein	0.01	1.66	CE	CE10
MAC_04294	alcohol dehydrogenase	-0.62	1.65	AA	AA3
MAC_07113	alpha/beta hydrolase fold domain containing protein	0.70	1.62	CE	CE1
MAC_02142	exo-beta-1,3-glucanase	0.53	1.52	GH	GH17
MAC_05737	N-acetylglucosaminidase	-0.28	1.43	GH	GH20
MAC_00345	endo alpha-1,4 polygalactosaminidase precusor	-0.27	1.40	GH	GH114
MAC_07994	FAD binding domain protein	0.48	1.38	AA	AA7
MAC_06700	hypothetical protein	0.01	1.17	GH	GH84
MAC_09703	chitinase 18-18	0.58	1.12	GH	GH18
MAC_00160	1,2-a-D-mannosidase	0.23	1.12	GH	GH47
MAC_02139	putative beta 1,3-galactosyltransferase polypeptide 1	0.29	1.06	GT	GT31
MAC_02485	chitinase	-0.92	-1.01	GH	GH18
MAC_05784	acetyl-hydrolase	-1.35	-1.05	CE	CE10
MAC_04394	exo-polygalacturonase, putative	-0.30	-1.06	GH	GH28
MAC_02090	Glycosyl hydrolase family 16 protein	0.08	-1.06	GH	GH16
MAC_06333	chitinase	-0.18	-1.09	CBM	CBM1
MAC_09392	Cel3b putative secreted beta-glucosidase	-0.76	-1.13	GH	GH3
MAC_06143	glycosyl transferase, putative	-0.70	-1.13	GT	GT32

Gene Symbol	Products name	log$_2$(Δ*Mavib-1* / WT)	log$_2$(OE / WT)	Enzyme	Family
MAC_00623	endoglucanase, putative	0.13	-1.34	AA	AA11
MAC_01220	NAD binding Rossmann fold oxidoreductase, putative	-0.41	-1.35	GH	GH109
MAC_02088	alpha-1,2 mannosyltransferase KTR1	-1.57	-1.66	GT	GT15
MAC_06282	cholinesterase	-0.27	-1.84	CE	CE10
MAC_06157	putative endochitinase CHI2	-0.09	-1.86	GH	GH18
MAC_08465	chitinase 18-3	-0.67	-1.88	GH	GH18

Table S8 DEGs related to nitrogen metabolism

Gene Symbol	Products name	log2(Δ*Mavib-1*/WT)	log2(OE/WT)
MAC_06383	aspartate carbamoyltransferase catalytic subunit	1.11	
MAC_06029	ornithine decarboxylase	1.03	
MAC_05125	S-adenosylmethionine decarboxylase proenzyme	1.09	
MAC_04872	urea active transporter	1.12	-1.25
MAC_05344	urea transporter	1.01	
MAC_07491	urease	1.00	
MAC_03493	nitrite reductase	1.36	
MAC_06321	NmrA family protein	-1.90	
MAC_09768	Amino-acid permease inda1	1.03	
MAC_05400	aspartate aminotransferase, putative	9.28	
MAC_06320	aspartic proteinase	-1.39	1.34
MAC_09277	glutamyl-tRNA (Gln) amidotransferase	-1.19	1.27
MAC_08898	glutamate decarboxylase, putative		3.65
MAC_09524	glutathione S-transferase		-1.23
MAC_05310	glutathione-dependent formaldehyde-activating		1.11
MAC_00229	amino acid permease, putative		3.14
MAC_09782	amino acid transporter arg-13		1.42
MAC_03365	amino acid transporter, putative		1.24
MAC_00316	Amino-acid permease inda1		4.02
MAC_04079	Amino-acid permease inda1		-1.55

MAC_08200	tryptophanyl-tRNA synthetase	5.86
